# Web-Based Interventions to Improve Mental Health, General Caregiving Outcomes, and General Health for Informal Caregivers of Adults With Chronic Conditions Living in the Community: Rapid Evidence Review

**DOI:** 10.2196/jmir.7564

**Published:** 2017-07-28

**Authors:** Jenny Ploeg, Maureen Markle-Reid, Ruta Valaitis, Carrie McAiney, Wendy Duggleby, Amy Bartholomew, Diana Sherifali

**Affiliations:** ^1^ School of Nursing Faculty of Health Sciences McMaster University Hamilton, ON Canada; ^2^ Aging, Community and Health Research Unit McMaster University Hamilton, ON Canada; ^3^ Department of Health, Aging and Society McMaster University Hamilton, ON Canada; ^4^ WHO Collaborating Centre for Primary Care and Health Human Resources McMaster University Hamilton, ON Canada; ^5^ Department of Family Medicine McMaster University Hamilton, ON Canada; ^6^ Department of Psychiatry & Behavioural Neurosciences Faculty of Health Sciences McMaster University Hamilton, ON Canada; ^7^ Program for Interprofessional Practice, Education and Research (PIPER) McMaster University Hamilton, ON Canada; ^8^ Faculty of Nursing University of Alberta Edmonton, AB Canada; ^9^ Innovations in Seniors' Care Research Unit Faculty of Nursing University of Alberta Edmonton, AB Canada; ^10^ McMaster Evidence Review and Synthesis Centre McMaster University Hamilton, ON Canada

**Keywords:** Internet, review, chronic disease, adult, caregivers

## Abstract

**Background:**

Most adults with chronic conditions live at home and rely on informal caregivers to provide support. Caregiving can result in negative impacts such as poor mental and physical health. eHealth interventions may offer effective and accessible ways to provide education and support to informal caregivers. However, we know little about the impact of Web-based interventions for informal caregivers of community-dwelling adults with chronic conditions.

**Objective:**

The purpose of this rapid evidence review was to assess the impact of Web-based interventions on mental health, general caregiving outcomes, and general health for informal caregivers of persons with chronic conditions living in the community.

**Methods:**

A rapid evidence review of the current literature was employed to address the study purpose. EMBASE, MEDLINE, PsychInfo, CINAHL, Cochrane, and Ageline were searched covering all studies published from January 1995 to July 2016. Papers were included if they (1) included a Web-based modality to deliver an intervention; (2) included informal, unpaid adult caregivers of community-living adults with a chronic condition; (3) were either a randomized controlled trial (RCT) or controlled clinical trial (CCT); and (4) reported on any caregiver outcome as a result of use or exposure to the intervention.

**Results:**

A total of 20 papers (17 studies) were included in this review. Study findings were mixed with both statistically significant and nonsignificant findings on various caregiver outcomes. Of the 17 included studies, 10 had at least one significant outcome. The most commonly assessed outcome was mental health, which included depressive symptoms, stress or distress, and anxiety. Twelve papers examined the impact of interventions on the outcome of depressive symptoms; 4 found a significant decrease in depressive symptoms. Eight studies examined the outcome of stress or distress; 4 of these found a significant reduction in stress or distress as a result of the intervention. Three studies examined the outcome of anxiety; 2 of these found significant reductions in anxiety. Other significant results of the interventions were seen in the outcomes of caregiver gain (ie, positive aspects of caregiving), knowledge, bonding, reduction of anger-hostility, and negative mood. Based on this review, it is not possible to determine which interventions were most effective since studies differed in their design, sample, and intervention. Study results suggest that Web-based interventions may result in reduced depressive symptoms, anxiety, and stress or distress among informal caregivers of adults with chronic conditions in the community.

**Conclusions:**

This is the first review assessing the impact of Web-based technologies on mental health, general caregiving outcomes, and general health for caregivers of adults with chronic conditions living in the community. Further rigorous research is needed that includes adequately powered studies examining the critical components of the intervention and the dosage needed to have an effect.

## Introduction

The number and proportion of adults living with chronic conditions is increasing globally. These adults are likely to live at home and rely on informal caregivers for support. Although informal caregivers experience rewards associated with caregiving, they also experience negative impacts such as burden, distress, and poor mental and physical health. Web-based programs may offer effective and accessible supports to improve caregiver outcomes. The purpose of this rapid evidence review was to assess the impact of Web-based interventions on mental health, general caregiving outcomes, and general health for informal caregivers of persons with chronic conditions living in the community.

Longer life expectancies and an aging population mean that an increasing number of adults are likely to develop chronic conditions and need complex care now and into the future. Globally, 68% of all deaths in 2012 were due to noncommunicable diseases such as cardiovascular disease, cancer, and diabetes [1]. In the United States in 2012, half of all adults had one or more chronic conditions and these were the leading cause of death and disability [2]. Increasingly, adults have multiple (two or more) chronic conditions (MCC). These individuals often experience poor health-related quality of life, are at increased risk for adverse events, and use more health services compared with those with a single condition [3]. For many individuals with chronic conditions, such as the estimated 228,000 Ontarians living with dementia, there are long-term health and social impacts of illness [4]. Unlike other health issues, chronic diseases are generally slow progressing, making their impact on society longstanding, expensive, and complex.

Most adults with chronic conditions live at home and rely on informal caregivers such as spouses, children, or other family or friends for support. Reasons for this reliance on informal caregivers include the limited hours of available home care, difficulty accessing services, and resistance to accepting outside help. Informal caregivers play a critical role in helping care recipients live with the complex issues of chronic conditions in their own homes rather than institutions [5,6].

In 2012, over one-quarter of Canadians aged 15 years and older (8.1 million individuals) provided care to a chronically ill, disabled, or aging family member or friend in the previous 12 months [7]. Caregiving tasks include providing transportation, housework and house maintenance, scheduling and coordinating medical appointments, help with finances, emotional support, and personal care. The median time spent on caregiving was 3 hours per week and for spousal caregiving, 14 hours per week [7]. The estimated economic contribution to the Canadian health care system of unpaid caregivers for older adults in 2009 was Can $25 billion [8]. Caregivers have multiple responsibilities in addition to their caregiver role, with 60% working at a paid job and 28% having children under the age of 18 years [7].

Although there are rewards associated with caregiving, caregivers have reported negative outcomes such as poor mental and physical health [7]. A meta-analysis of 84 studies found that caregivers have statistically significant higher levels of stress and depression and lower levels of subjective well-being, physical health, and self-efficacy compared with noncaregivers [9]. Furthermore, a prospective population-based cohort study found that, among older caregivers, a state of mental or emotional strain was associated with a 63% higher risk of mortality compared with noncaregivers [10]. Although these conclusions need to be carefully considered with respect to health care context and current understanding, it does illuminate the burden that caregivers may face. Caregiving may also result in disruptions to work routines [7]. Given the negative impacts of caregiving, caregivers may require support to ensure their own well-being.

Many interventions designed to support informal caregivers have been evaluated and have the potential to improve caregiver outcomes. A recent systematic review of systematic reviews on interventions for caregivers of persons with chronic conditions found that education and support interventions improved caregiver quality of life [11]. Most caregiver support interventions are offered face to face. eHealth interventions may offer efficient, less costly, and more accessible ways to provide education and support to informal caregivers [12]. Web-based interventions may be more easily accessed by caregivers from their own homes or workplaces.

There has been a rapid growth in the use of the Internet in the past 15 years, with 84% of American adults and 88.5% of Canadians using the Internet [13-15]. Although variable rates occur between socioeconomic and age groups, Canada leads Web-based engagement, with the average Canadian spending over 41 hours on the Web each month [13]. The fastest growing demographic for Internet use is in adults aged 55 years and above [13]. Furthermore, 80% of Internet users go on the Web to seek health information [5]. The Internet has become a valuable tool to provide information and connect people with others who are experiencing similar health issues and “enables new pathways for patients to find and help each other” (p. 6) [5]. In addition, caregivers of people with chronic disease often seek information and support on the Web [5]. Twenty-six percent of adult caregiver Internet users went on the Web to find other individuals who were caring for loved ones [5]. One of the groups most likely to look on the Web for health information comprises adults who have provided unpaid caregiving within the past 12 months [5]. Given this high Internet use and the potential for gaining valuable health information and support, Web-based interventions may play an integral role in decreasing caregiver burden and distress and improving their health outcomes.

Multiple systematic or other reviews of technology interventions (eg, the Web and telephone) to support informal caregivers of adults in the community have been noted in the literature [16-25]. These reviews examined the impact of the interventions on a variety of caregiver outcomes such as mental health (eg, stress, depressive symptoms, and anxiety), burden, quality of life, and social support. Most reviews concluded that there were mixed findings of the impact of the interventions; studies demonstrated positive, none, or negative effects on caregiver outcomes. Overall, mixed results were reported, due primarily to a combination of limited methodological quality (eg, weak design and small sample sizes).

Only 3 reviews focused specifically on Web-based interventions designed for caregivers [18,23,25]. Most reviews were specific to certain caregiving groups, primarily caregivers of persons living with dementia [16-22], cancer [23], and stroke [24]. One review included caregivers of persons with both acute and chronic conditions, as well as a study of caregivers of children [25]; it is not clear how similar or different the experiences, impacts, or effectiveness of technology interventions are among these different subgroups. Two reviews included only randomized controlled trials (RCTs) and controlled clinical trials (CCTs) [19,24]. Other reviews included studies of lower methodological quality than RCTs and CCTs, such as pre-post design studies. Many reviews examined the impact of Web-based technologies on specific outcomes such as caregiver stress [25] or burden [17]. None of the reviews focused specifically on the impact of Web-based interventions on mental health, general caregiving outcomes, and general health for informal caregivers of adults with chronic conditions living in the community.

## Methods

### Design

We used a rapid evidence review approach [26]. These reviews are a streamlined alternative to standard systematic reviews and meet the needs of faster-paced health care decision-makers [27]. This approach was well suited to the current work as the review was conducted in response to a request from policy decision makers for a synthesis of knowledge related to the impact of Web-based interventions on caregiver outcomes. Consistent with rapid evidence review approaches, we limited the review in selected ways, specifically (1) including only RCTs and CCTs representing the highest quality of study design, (2) including only papers published in English and excluding conference abstracts and dissertations, (3) omitting personal communication with experts as a search strategy, and (4) not including a quality assessment of the included studies.

### Search Strategy

A peer-reviewed search strategy was developed by two research librarians at McMaster University. EMBASE, MEDLINE, PsychInfo, CINAHL, Cochrane, and Ageline were searched covering all studies published from January 1995 to July 2016. Reference lists of systematic reviews were searched for relevant studies not captured by our search. Once the search was completed and uploaded, duplicates were removed, and the citations were uploaded to a secure Web-based platform. More detailed information about the search terms is available in Multimedia Appendix 1.

### Selection of Studies

The titles and abstracts of papers were reviewed by two members of the synthesis team who collectively have 30 years of experience following Cochrane systematic review methods; any article marked for inclusion by either team member went on to full text rating. Full text inclusion was done independently by two people. All disagreements were resolved through discussions rather than relying on a particular level of kappa score to indicate when discussions were no longer necessary. The inclusion results were reviewed by a third person who was also a member of the synthesis team.

For each study, one team member completed full data extraction using electronic forms. A second team member then verified all extracted data; disagreements were resolved through discussion or third party consultation when consensus could not be reached. For each study, review team members extracted data about the population, the study design, the intervention, and the results for outcomes of interest using a standardized data extraction form. Details of the interventions were extracted based on the Template for Intervention Description and Replication Checklist [28].

Papers selected for this review were any study which included a Web-based modality to deliver an intervention (either stand alone or multi-modal) and met the following criteria: (1) included informal, unpaid adult caregivers of adults (≥ 18 years of age) who were living in the community with a chronic condition or health issue; (2) was either an RCT or a CCT; and (3) reported on any caregiver outcome as a result of use or exposure to the intervention. RCTs are trials where the groups compared were established by random allocation, whereas CCTs are trials where the method of allocation of participants to groups was not necessarily random [29].

## Results

### Search Results

**Figure 1:**
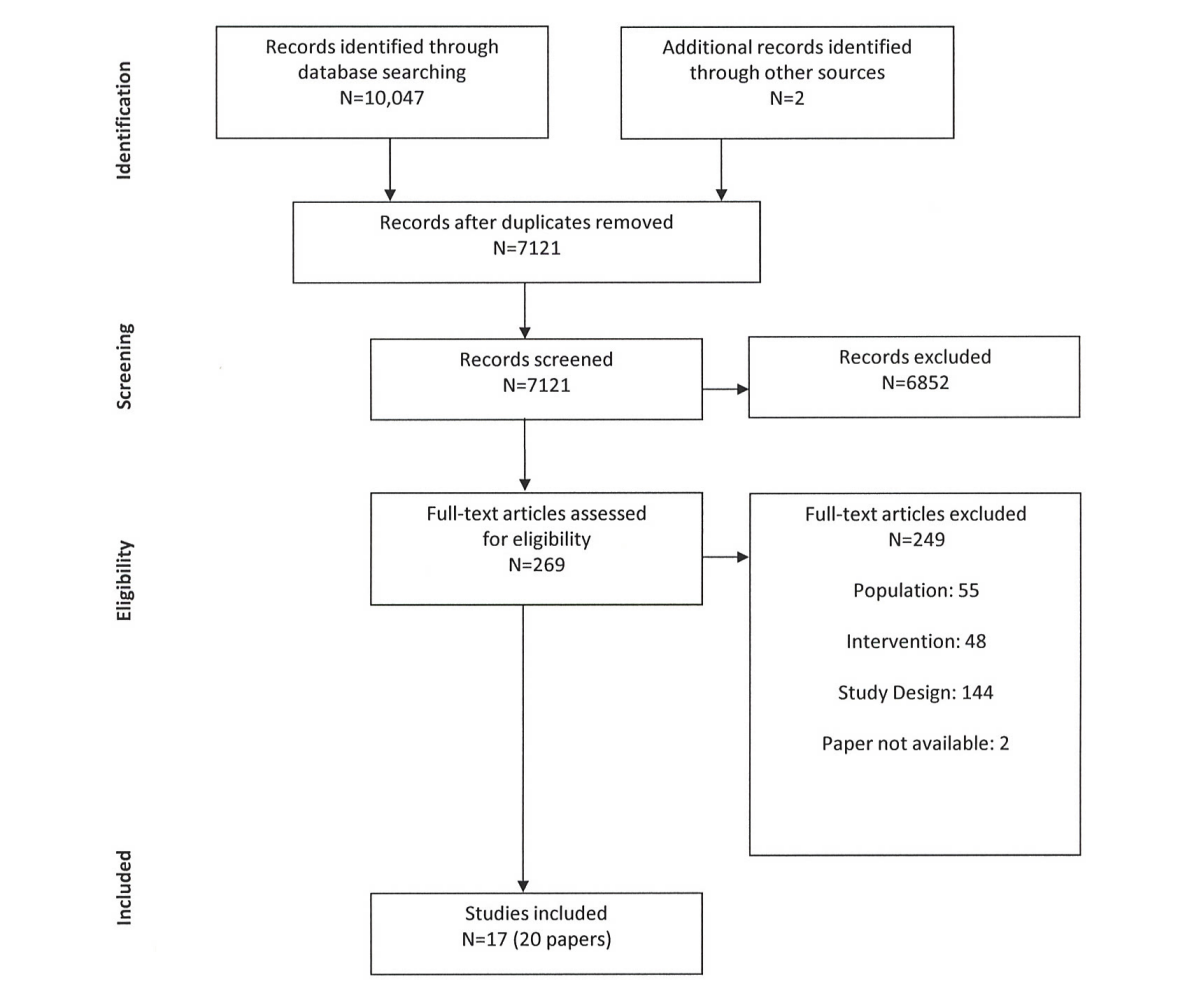
Study flow diagram.

The database search identified 10,047 journal articles and a further 2 articles were identified from other sources (see Figure 1). After duplicates were removed, 7121 articles remained. After additional screening, 6852 articles were excluded, leaving 269 articles for assessment of eligibility. From these, 249 additional articles were then excluded resulting in 20 papers from 17 unique studies that met the inclusion criteria. Of the 20 papers, 19 reported caregiver outcomes. For 1 study, 2 papers reported on caregiving outcomes [30,31], and 1 paper reported on methods [32]. See Multimedia Appendix 2 for a detailed description of the included studies (19 papers that include caregiver outcomes are presented). Of the 17 studies included in this review, 11 were RCTs, 5 were CCTs [12,33-36], and 1 was a combination of both RCT and CCT [37]. Sample size ranged from 19-299 caregivers. Overall, 11 studies were completed in the United States [30,31,33,35,36,38-43], 2 in Canada [34,44], and 1 in both the Netherlands and France, respectively [45,46]. The remaining studies were completed across several countries including the Netherlands and the United Kingdom [47]; Germany, Netherlands, and Belgium [37]; Puerto-Rico, United States, and Mexico [12]; and the United Kingdom, Spain, and Greece [48].

Interventions were targeted at diverse groups of caregivers including caregivers of persons with (1) Alzheimer disease, dementia, or neurodegenerative disease [12,34,37-39,44-48]; (2) cancer [30,31]; (3) stroke [41-43]; (4) heart transplant [33]; (5) traumatic brain injury [40]; (6) chronic disease [36]; and (7) at least one health or safety concern, regardless of their diagnosis [35]. The mean age of caregiver participants in studies reporting the mean (n=16) was 57.72 years. Of the remaining 3 studies, one reported 48% of caregivers were aged 50+ years, another reported 39.8% of caregivers were aged 51+ years, and one stated that >50% of caregivers were aged 55 years and above. Of the 14 studies that reported on caregiver gender, 74.29% of caregivers were female. Two studies specifically targeted employed caregivers [35,36].

For 17 of the 19 studies that included outcome data, the comparison was between the intervention and a control group; 2 of the CCTs [34,35] compared 2 different interventions. Control groups (described in Multimedia Appendix 2) were (1) provided with usual care only [37,41,43,46,48], (2) provided with usual care plus minimal information (ie, e-bulletins, pamphlets, and newsletters) [12,30,31,39,40,42,45], or (3) wait listed 30-120 days to receive the intervention [38,47]. In one study, the control group consisted of people who did not have access to the website who were part of other longitudinal studies [33]. Three CCTs compared variations with their intervention; one compared being in a Web-based chat support group with a Web-based videoconferencing support group [34], whereas another gave enrolled participants the option of “high tech” or “low tech” support due to concerns over equal access in a work environment [35]. The last CCT used a control group of nonactive participants who posted or read fewer than 4 messages on the online support group [36].

Many caregiver outcomes were assessed across the studies, as indicated in Multimedia Appendix 3. The most common outcomes included (1) mental health outcomes such as depressive symptoms (n=12), stress or distress (n=8), and anxiety (n=3); (2) general caregiving outcomes such as burden (n=5), mastery or self-efficacy (n=5), and social support (n=4); and (3) general health outcomes such as quality of life (n=6) and overall health (n=4).

Based on the results of the literature search, we classified the study interventions into the following categories that were adapted from Jackson et al [16]: (a) single component interventions (information/education) and (b) multicomponent interventions including: (1) information/education plus peer psychosocial support; (2) information/education plus professional psychosocial support; (3) information/education plus peer and professional psychosocial support; (4) information/education plus monitoring plus professional psychosocial support; and (5) monitoring plus peer and professional psychosocial support.

### Single Component Interventions: Information or Education

Three Web-delivered interventions were RCTs categorized as information or education only interventions [38-40]. The intervention, Caregiver’s Friend: Dealing with Dementia, included text and videos to support employed caregivers of persons with dementia [38]. The iCare Stress Management program aimed to teach coping skills to caregivers of persons with dementia [39]. The Brain Injury Partners website provided text and video to teach advocacy skills to caregivers of persons with brain injury [40].

Two studies found statistically significant lower stress scores as a result of receipt of the intervention. Beauchamp et al [38] found a reduction in stress after 30 days using a two-question scale (*P*<.001); Kajiyama et al [39] found a reduction in stress using the Perceived Stress Scale (PSS) following 3 months of the intervention (*P*=.02). Beauchamp et al found a small reduction in depressive symptoms in the intervention group compared with the control group (*P*=.009) using the Center for Epidemiological Studies Depression Scale (CES-D). Kajiyama et al also assessed depression using the CES-D but did not find a statistically significant reduction in depressive symptoms (*P*=.26). One study found statistically significant lower scores for anxiety and caregiver strain, as measured by the State-Trait Anxiety Inventory (*P*=.03) and Caregiver Strain Instrument (*P*=.03), respectively, as well as improved scores for self-efficacy in the intervention compared with control group (*P*=.02) [38]. Finally, a study using Web-based training in family advocacy found statistically significant higher scores for knowledge of (*P*=.03), ability to apply (*P*<.001), and intention to use (*P*<.001) advocacy skills but no difference in satisfaction with life (*P*=.05) as a result of the intervention [40]. Overall, the three single component Web-based information or education RCT interventions demonstrated mixed findings, with some improvements in mental health and caregiver outcomes reported.

### Multicomponent Interventions: Information or Education Plus Peer Psychosocial Support

Two studies, both RCTs, used a combination of information or education and peer psychosocial support offered through the Web [46,47]. The Diapason program, aimed at caregivers of persons with Alzheimer disease, consisted of a website with 12 educational sessions and a private forum for caregivers to interact with peers to share experiences [46]. The Skills Training and Reskilling (STAR) course was a Web-based portal containing eight modules to support caregivers of persons with dementia in the Netherlands and the United Kingdom [47]. STAR also offered linkages to communities of other caregivers via Facebook.

In both studies, there were no significant differences between groups on caregiver burden, as measured by the Zarit Burden Interview (ZBI) (*P*=.74) [46] and a single question to assess burden (not reported) [47]. In the Diapason program, no significant differences were found at 6 months between the intervention and control groups in caregiver stress (PSS; *P*=.98), self-efficacy (Revised Scale for Caregiving Self-Efficacy [RSCS], *P*=.52), reaction to problem behavior (Revised Memory and Behavior Problems Checklist [RMBPC]; *P*=.66), depressive symptoms (Beck Depression Inventory; *P*=.56), or self-perceived health (Nottingham Health Profile; not reported). There were no differences between groups at 6 months on the outcomes of knowledge (about Alzheimer disease), stress, self-efficacy (for coping with the illness), or quality of relationship (between caregiver-person with dementia), as measured by a Visual Analog Scale.

In the STAR program [47], there were statistically significant changes from baseline to 2-4 months after the intervention between the intervention and control groups on empathy subscales, as measured by the Interpersonal Reactivity Index (*P*=.003): intervention participants reported that they felt less distressed in tense situations, had more empathy for the well-being of others, and were better able to understand others’ situations and actions. There were no statistically significant differences between groups on quality of life (*P*=.97). However, there was a negative effect noted following the completion of the course on one’s sense of competence (*P*=.02), where course participants felt less competent to fulfill caregiving roles after the course [47]. Overall, the two multicomponent interventions including information or education plus peer psychosocial support demonstrated minimal impact on caregiver outcomes.

### Multicomponent Interventions: Information or Education Plus Professional Psychosocial Support

One RCT comprised an intervention called Mastery over Dementia [45] that included a Web-based course of 8 lessons over 5 to 6 months, homework following each lesson with feedback provided electronically by a coach (psychologist), as well as a final booster session guided by the coach. The course was designed to reduce caregiver depression and anxiety. The intervention group showed statistically significant lower depressive symptoms (CES-D 20) (*P*=.02) and anxiety (Hospital Anxiety and Depression Scale; *P*=.008) from baseline to 5-6 months, noting the effect size for anxiety was moderate (0.48) and small for depressive symptoms (0.26) [45].

### Multicomponent Interventions: Information or Education Plus Peer and Professional Psychosocial Support

Ten separate studies (11 papers total) described the impact of multicomponent interventions that included a combination of information or education, plus peer and professional psychosocial support. Two papers reported on an RCT of a Web-based lung cancer information, communication, and coaching system for caregivers called Comprehensive Health Enhancement Support System (CHESS) [30,31]. CHESS comprised information (eg, frequently asked questions, resource directory, and Web links), communication services (eg, discussion board monitored by a professional facilitator and online groups for caregivers), and coaching and training services (eg, data about the patient’s health status, decision aids, and coping supports for distress). Following 6 months, the intervention group had statistically significant lower levels of burden, measured with the Caregiver Quality of Life-Cancer scale (CQOLC) burden scale (*P*=.02) and negative mood, measured by the Short Version Profile of Mood States (*P*=.006) than the control group who received only access to websites on lung cancer [30]. However, there were no differences between groups on disruptiveness (ie, the degree to which caregiving tasks interfere with regular daily routines), as measured by the Disruptiveness subscale of the CQOLC (*P*=.15) [30]. In a secondary analysis of the data, Namkoong et al found that the CHESS intervention group perceived higher bonding with other caregivers than the control group, as measured by a 5-item scale (*P*=.04) and that bonding was positively associated with caregivers’ coping strategies [31].

Two papers reported on an RCT of Caring~Web [41,43], a Web-based intervention that offered education and support for caregivers of stroke survivors. The Caring~Web program provided access to linked websites about stroke and caregiving, educational information specific to caregivers’ needs, an email forum linked to a nurse specialist and rehabilitation team members, and a nonstructured email discussion group of caregivers facilitated by the nurse [41,43]. These studies reported that after 1 year, there were no significant differences between the intervention and control groups on depressive symptoms (CES-D; *P*=.48), life satisfaction (Satisfaction with Life Scale; *P*=.90) [41], caregiver self-rated health as measured by the Multidimensional Functional Assessment of Older Adults, and receipt of emotional support or physical help from family or friends [43].

Using an RCT design, Smith et al [42] adapted the Caring~Web intervention to include a professional guide to facilitate educational modules, 11 educational videos, and chat room sessions. Caregivers in the intervention group reported statistically significant lower depressive symptoms on the CES-D scale (*P*<.01) than the control group [42]. However, there were no differences between groups on mastery, self-esteem, or social support when assessed with the Mastery Scale, Self-Esteem Scale, and the MOS Social Support Survey, respectively [42].

Four studies reported on Web-based supports for caregivers of persons with dementia or neurodegenerative disease [12,34,44,48]. Marziali and Donahue [44] used an RCT to assess the impact of a website, Caring for Others, aimed at caregivers of persons with neurodegenerative disease such as Alzheimer, stroke, and Parkinson. The website included links to information, email, and a videoconferencing link. The videoconferencing link supported participation in a 10-session psychosocial support group, followed by 12 additional online sessions facilitated by a group member. After 6 months, there were no statistically significant reductions following the intervention in depressive symptoms (CES-D), health-related quality of life measured by the Health Status Questionnaire, stress experienced in relation to performing activities of daily living (ADL) or instrumental ADL (IADL) for the care recipient, reaction to problem behaviors (RMBPC), or social support as measured by the Multidimensional Scale of Perceived Social Support (MDSPSS). When the authors combined the two stress measures (ADL and IADL) and managing difficult behaviors, they found a statistically significant decline in stress in the intervention group (*P*<.004) [44].

In a multi-site, CCT study, Marziali and Garcia [34] compared two interventions: (1) a Web-based chat support group plus 6 dementia care educational videos and (2) a Web-based videoconferencing support group facilitated by a health professional. After 6 months, both interventions showed significant improvements in self-efficacy (RSCS; *P* ≤.04) compared with baseline measures. The videoconferencing support group showed significantly lower distress scores associated with managing deterioration in mental function of the care recipient, measured with the Functional Autonomy Measurement System (*P*<.02) and a greater improvement in mental health, as measured by the Health Status Questionnaire (*P*<.02). However, the Web-based chat group had lower distress scores related to managing IADL of the care recipient than the videoconferencing group (*P*<.02). There were no differences between groups on depressive symptoms (CES-D) or social support (MDSPSS) [34].

Using a CCT design, Pagan-Ortiz et al [12] examined the impact of a website, Cuidate Cuidador, for Spanish-speaking caregivers in three countries: Puerto-Rico, United States, and Mexico. The website included information about Alzheimer disease and related dementias, strategies for managing dementia-related behaviors, a section to interact with other caregivers, and an Ask an Expert resource section. After 1 month, there were no statistically significant differences between the intervention and control group (who received educational materials) on depressive symptoms (CES-D; *P*=.93), sense of self-mastery (Personal Mastery Scale; *P*=.17), sense of social support (Lubben Social Network Scale; *P*=.98), or caregiver burden (ZBI; *P*=.77) [12].

Torkamani et al [48] used an RCT to test the impact of a computerized platform called ALADDIN (A technology pLatform for the Assisted living of Dementia elDerly INdividuals and their carers) in three countries: United Kingdom, Spain, and Greece. ALADDIN had four key features: (1) ALADDIN TV to provide information and educational material; (2) a social networking forum to connect with other carers; (3) a My Tasks distant monitoring feature where caregivers completed questionnaires about their own health and that of the care recipient, and responses were monitored by clinicians who intervened as needed; and (4) a contact us feature that alerted a request from a clinical site for contact [48]. The ALADDIN group had higher quality of life than the control group at 6 months, as measured by the EuroQol (*P*=.03); there was no difference in quality of life as measured by the Quality of Life scale (*P*=.56). There were no statistically significant differences between the ALADDIN intervention group and control group on burden or distress, as measured by the ZBI (*P*=.19) and the Neuropsychiatric Inventory (*P*>.05); impact on depressive symptoms was not assessed due to missing data.

One study in this category specifically tested Web-based interventions to support employed caregivers [36]. This CCT compared two types of interventions for caregivers of persons with chronic disease: an online support group professionally facilitated by a clinical nurse specialist, a moderated or peer-directed support group, and a control group which comprised nonactive participants of an online group (posted and viewed less than 4 messages) [36]. At 12 weeks, caregivers in both the professionally facilitated and moderated or peer-directed groups had statistically significant lower depressive symptoms (CES-D; *P*=.04 and *P*=.03, respectively) and higher quality of life, as measured by the Caregiver Quality of Life Index than the nonactive participants (*P*=.01, *P*=.008) [36]. There were no differences between intervention groups and the control group on caregiver strain (CSI) nor were there differences between the two intervention groups on depressive symptoms (*P*=.52) or quality of life (*P*=.71).

Another CCT examined the impact of a Web-based intervention for caregivers of heart transplant recipients compared with people without access to the website [33]. The intervention included “HeartNet” website comprising information on transplant-related health issues, stress and medical regimen workshops, access to electronic communication with the transplant team, and monitored discussion groups. Study participants were assessed 4 to 6 months later and the intervention group caregivers had statistically significant lower anger-hostility symptoms (*P*=.03), as measured by the Symptom Checklist-90 subscales for anger-hostility, compared with the control group; however, depressive symptoms (*P*>.05) and anxiety scores (*P*=.05) were not different between the groups [33].

Overall, the 10 multicomponent interventions involving information or education plus peer and professional psychosocial support showed mixed findings. Some interventions had a positive impact on mental health, caregiving, and general health outcomes, whereas others showed no differences in outcomes between intervention and control groups.

### Multicomponent Interventions: Information or Education Plus Monitoring Plus Professional Psychosocial Support

The CCT in this category was the second study to specifically test Web-based interventions to support employed caregivers [35]. Working caregivers received access to either an online caregiver support group moderated by a geriatric or psychiatric nurse, Web-based information and Web-based consultation with a geriatrician, or a remote monitoring system for the older adult to wear at home. The monitoring system provided Web-based status reports and email or pager alerts when activity parameters of the care recipient were exceeded. Following 6 months, results suggested that caregivers in both intervention groups experienced reduced caregiver stress, increased morale, and increased worker productivity, but given the small sample size (N=19), statistical significance was not assessed.

### Multicomponent Interventions: Monitoring Plus Peer and Professional Psychosocial Support

One study (completed in three countries using both RCT and CCT designs) used a combination of the Web plus telephone as the intervention modality compared with usual care that included home care [37]. The Rosetta system involved the home installation of sensors and cameras to support the person with dementia with navigation, an early detection surveillance system that warned carers of changes in day-to-day patterns and activities, as well as a monitoring system to alert in times of emergencies (ie, falls). At the end of the study, approximately 4 months post baseline assessment, there were no significant differences between caregivers who used Rosetta and the usual care group (eg, home care services) on the outcomes of quality of life (*P*=.37), as measured by the Quality of Life in Alzheimer Disease Scale and feelings of competence (*P*=.11) using the Short Sense of Competence Questionnaire.

### Website Use and Influence on Outcomes

Of the 17 included studies, 11 provided some information about the use of the intervention website (see Multimedia Appendix 4 for details), 5 assessed the impact of website usage on outcomes, 1 reported a significant correlation between time spent on the website and a composite outcome measure [38], 1 found greater website use was correlated with greater improvement on a quality of life domain [33], 1 found a significant correlation between more time on the website and change in knowledge [40], and 2 found no association between website use and outcomes [39,46].

### Pragmatic Quality Review of Studies

We used a pragmatic approach consistent with rapid evidence review approaches to identify higher quality studies (without doing a more comprehensive quality assessment which is planned for future work). First, we included only those studies using an RCT design (and excluded the 6 studies using a CCT design). Next, we excluded studies that reported their work as pilot studies; this left us with 7 studies. Finally, we excluded studies that did not provide a sample size calculation; this left us with 2 studies (4 papers) [30,31,41,43]. These two RCTs have been described earlier as the CHESS [30,31] and the Caring~Web studies [41,43] (see Multimedia Appendix 2). Both studies involved multiple components, specifically information or education plus psychosocial support provided by both peers and professionals. The CHESS study included 285 patient-caregiver dyads of persons with advanced nonsmall cell lung cancer and the Caring~Web study included 73 caregivers of first-time stroke survivors. The outcomes of these two higher quality studies were mixed with both statistically significant and nonsignificant findings, similar to the overall review results. The CHESS study found reduced caregiver burden and negative mood, as well as improved bonding but no group differences on coping after 6 months [30,31]. The Caring~Web study found no group differences in depressive symptoms, satisfaction with life, health, emotional support, or physical help after 1 year [41,43]. This analysis of 2 higher quality studies suggests that further research is needed to understand the impact of such interventions.

## Discussion

### Principal Findings

This is the first known review assessing the impact of Web-based technologies, designed for use by caregivers of adults with chronic conditions living in the community, on caregiver outcomes, specifically mental health, general caregiving outcomes, and general health. This review included only RCTs and CCTs, constituting the most rigorous designs. The findings across studies were not comparable due to variations in design, sample, and interventions. In this review, more than 35 different measures were used to assess intervention outcomes, with the most commonly used tool being the CES-D to assess depressive symptoms (see Multimedia Appendix 3). This heterogeneity of outcome measures limits the ability to conduct comparison across studies. Five studies used nonvalidated tools or items to assess outcomes. The follow-up periods ranged from 1-12 months, with 9 studies having a somewhat limited follow-up of less than 6 months. The included papers provided minimal description of usual care.

Results show a mix of statistically significant and nonsignificant findings on various outcomes of the interventions (see Multimedia Appendix 2). The most important results were related to the positive impact of interventions on mental health: (1) 4 out of 12 studies examining the outcome of depressive symptoms found a statistically significant decrease in depressive symptoms in the intervention group, (2) 4 out of 8 studies examining the outcome of stress or distress found a significant reduction in stress or distress for the intervention group, and (3) 2 of 3 studies that examined the outcome of anxiety found a significant reduction due to the intervention.

General caregiving outcomes were also commonly assessed with mixed findings: (1) 2 of 5 studies found improved mastery or self-efficacy, (2) 1 of 5 studies found reduced burden, (3) 1 of 2 studies found reduced strain, (4) none of 3 studies found improved reaction to care recipient problem behaviors, (5) neither of 2 studies found improved coping as a result of the intervention, and (6) none of 4 studies found improvements in social support. In terms of general health (1) 2 out of 6 studies found an improvement in quality of life, (2) none of 4 studies found improved overall health, and (3) none of 2 studies found improved life satisfaction. Individual studies reported on a variety of other outcomes with mixed findings (see Multimedia Appendix 3).

There were mixed findings across types of interventions (Table 1). Of the 3 studies involving Web-based information or education only, 1 study found primarily positive impacts on mental health and general caregiving, one study found no impact on general health, and a third study found both positive and no effects on mental health outcomes and no impact on general caregiving. Of the 2 studies examining information plus peer support, one found no effect on general caregiving, general health, and mental health outcomes, and a second study found some general caregiving and mental health outcomes were positively impacted, and others had no change. The one study involving information or education and professional support demonstrated improved mental health outcomes. Studies including monitoring as part of the intervention found no impact on quality of life [37] or stress [35]. Ten studies (11 papers) included a combination of information or education, peer and professional support. Five of these 10 studies examining a mental health outcome demonstrated an improvement. Only 2 of 8 studies found an improvement in general caregiving outcomes, and 2 of 6 studies found improvement in general health.

Six of the interventions had components that were tailored to the unique needs of caregivers [30,35,38,42,47,48]. Tailoring included, for example, an assessment of baseline knowledge and confidence to help guide the caregiver on which educational module to start with [47]. All but one of these studies demonstrated positive impacts on either mental health, caregiving, or general health outcomes. Previous studies have shown the value of tailoring interventions for behavior change [49].

Two recent systematic reviews of caregiver interventions have compared the impact of Web-based or remote interventions to other types of interventions [16,50]. A systematic review of four types of social support interventions (ie, befriending and peer support, family support and social network, support group, and remote interventions including the Web and phone) for caregivers of persons with dementia concluded that there was insufficient evidence on which intervention type works best to improve social support [50]. However, the authors noted that all intervention types resulted in positive but inconsistent effects on caregiver outcomes such as depression, burden, and quality of life and that multicomponent interventions were more effective than single component interventions. Another review of interventions for caregivers of persons with dementia found that combined telephone and Web-based interventions were more effective than telephone or Web-based interventions alone on outcomes such as depression, burden, and self-efficacy [16]. Both reviews concluded that included studies had important methodological limitations and sources of bias and that further research is warranted to improve the evidence base in this area.

Although there were very few studies (n=17) that met the inclusion criteria and they had mixed findings and diverse types of interventions, this rapid evidence review found that Web-based interventions may result in improvements in mental health outcomes such as depressive symptoms, anxiety, and stress. Furthermore, Web-based interventions that are tailored to the unique needs of caregivers may hold promise for improving caregiver outcomes.

Whereas we did not conduct a formal quality assessment of included studies, our data extraction revealed a number of methodological limitations, similar to other reviews of Web-based interventions to support caregivers [23]. Six studies had very small numbers of study completers (n<50 participants) [12,35,37,42,44,46], which may have resulted in nonsignificant findings; in 2 of those studies which were evaluating feasibility of the intervention, some outcomes could not be evaluated due to small sample size [35,37] (see Multimedia Appendix 2).

Some studies reported high dropout rates of caregivers and limited use of the Web-based intervention; for example, Blom et al [45] reported that almost 40% of caregivers dropped out of the intervention before the end of the study mainly due to lack of time or energy, use of other services, the intervention being less suitable, or a change in the care recipient status. Nonuse in the intervention group could have an impact on the dosage or the amount of the intervention that was needed to have an impact on outcomes. In a review of Web-based interventions for caregivers of persons with cancer, authors identified the limited information available about the dose of Web-based interventions [23]. It is recommended that studies describe the critical components of the intervention and the dosage needed to have an effect [51]. Furthermore, Web-based program developers should consider acceptability of the program to potential users through, for example, codesign efforts.

**Table 1:** Outcomes by intervention types.

Outcomes		Intervention Types																
		Information or education only n=3			Information or education plus peer support n=2			Information or education plus professional support n=1			Information or education plus peer and professional support n=11			Information or education plus monitoring and professional support n=1			Monitoring plus peer and professional support n=1	
		Sig^a^	NS^b^	Sig		NS	Sig		NS	Sig		NS	Sig		NS	Sig		NS
**Mental health outcomes^c^**																			
	Decreased depressive symptoms	[38]	[39]			[46]	[45]			[42] [36]		[41] [44] [34] [12] [48]^d^ [33]						
	Reduction in stress or distress	[38] [39]		[47]		[46]				[34]		[44] [48]			[35]^d^			
	Reduction in anxiety	[38]					[45]					[33]						
**General caregiving outcomes^e^**																			
	Improved mastery or self-efficacy	[38]				[46]				[34]		[42] [12]						
	Reduced caregiver burden					[46] [47]				[30]		[12] [48]						
	Reduced strain	[38]										[36]						
	Improved reaction to care recipient problem behaviors		[39]			[46]						[44]						
	Improved coping		[38]									[31]						
	Increased social support											[42] [34] [12] [44]						
**General health outcomes**																			
	Improved quality of life		[39]			[47]				[36] [48]^f^		[44] [48]^f^						[37]
	Improved overall health		[40]			[46]						[43] [34]						
	Improved life satisfaction		[40]									[41]						
**Other outcomes^h^**		See details below																		

^a^Sig: significant.

^b^NS: Not significant.

^c^Other mental health outcomes: anger-hostility [33]^a^, negative mood [30]^a^.

^d^Unable to evaluate.

^e^Other general caregiving outcomes: caregiver gain [38]^a^, emotional support [43]^b^, empathy [47]^a^, reaction to problem behavior and stress measures combined [44]^a^, self-esteem [42]^b^; and sense of competence [37,47]^b,g^

^f^Separate tools used for measurement.

^g^Statistically significant negative result.

^h^Other outcomes: ability to apply advocacy skills [40]^a^, attitudes [47]^b^_,_ bonding [31]^a^, disruptiveness [30]^b^, increased knowledge [40,47]^a^, intention to use advocacy skills [40]^a^, perspective [47]^a^, physical help [43]^b^, Visual Analog Scale (knowledge, stress, self-efficacy, and quality of relationship) [46]^b^.

Overall, there was great heterogeneity in study design (ie, population characteristics, sample size, and randomization) as well as intervention, which may explain some of the variability in the outcomes. However, without conducting further analysis (eg, regressions to control for these variations), any thoughts on the cause of the differences in outcomes would be speculative. Our research team is currently updating the literature review and plans to conduct a meta-analysis, where we will examine differences in outcomes based on factors such as study design and study quality.

Study results suggest that further research is needed in this relatively new area. First, rigorous, well designed, and adequately powered studies are needed to test the impact of Web-based interventions for caregivers. Research should more carefully describe and assess the components and dose of the intervention that are needed to result in improved outcomes. Future research should examine the impact of Web-based interventions on different groups of caregivers [23] and the role of tailoring interventions. Since the impact of caregiving varies among caregivers, it is likely that the impact of interventions aimed at reducing the negative effects of caregiving will vary. Research is also needed to describe the impact of the interventions on caregivers of persons with not just one but multiple chronic conditions, given the high prevalence of multimorbidity among older adults and the complexity experienced by caregivers in supporting these individuals [52].

### Study Strengths and Limitations

There are a number of strengths of this review. First, there was a very broad search for relevant papers in 6 key databases and over 10,000 records were reviewed based on a rigorous search strategy developed by two librarians. Second, only studies with the strongest designs, RCTs, and CCTs were included. Third, even though this was a rapid evidence review, a rigorous review approach was maintained, including (1) two expert librarians developed the search strategy; (2) citations in relevant systematic and narrative reviews were assessed for possible papers for inclusion; (3) titles and abstracts of papers were reviewed by two team members; (4) full text inclusion was conducted independently by two people with experience in conducting high quality systematic reviews, and included studies were reviewed by a third person who was also a member of the synthesis team; and (5) one team member completed full data extraction, and this was verified by a second person.

There are also a number of review limitations. First, only English language papers published in peer reviewed journals were reviewed. Second, the search did not include contact with experts, so some relevant studies may not have been included. Third, a formal quality assessment of the included studies was not conducted. This would have provided more detail on some of the methodological strengths and limitations of included studies. This area of study, Web-based interventions for caregivers, is not yet a robust field, and whereas we tried to ensure the highest possible rigor of studies by including only RCTs and CCTs, there was certainly variability in the degree of previous work completed on these interventions. Some interventions were clearly in the pilot phase, whereas others were further along in development and testing. Future research of such interventions should consider fidelity to the intervention and the implementation process.

### Conclusions

Web-based interventions to support caregivers of persons with chronic conditions are a relatively new but promising addition to the currently offered caregiver supports. This rapid evidence review suggests that Web-based interventions may result in improved mental health, general caregiving, and general health outcomes, although effects and improvements on study outcomes varied. Based on this review, it is not clear which types of Web-based interventions are most effective and for whom. Further work needs to be done and our team plans to complete an update of the literature and meta-analysis of the data to further add to this discussion. Important potential benefits of Web-based interventions are that they may be less costly than those involving face-to-face support from professionals, and they may be more accessible to caregivers. However, further rigorous research is needed that includes adequately powered studies examining the critical components of the intervention and the dosage needed to have an effect.

## Appendix

Multimedia Appendix 1 Search terms.

Multimedia Appendix 2 Characteristics of outcome studies.

Multimedia Appendix 3 Outcome measures.

Multimedia Appendix 4 Website use and influence on outcomes.

## Abbreviations

ADL: activities of daily living
ALADDIN: a technology pLatform for the Assisted living of Dementia elDerly INdividuals and their carers’
CCT: controlled clinical trial
CES-D: Center for Epidemiological Studies Depression Scale
CHESS: Comprehensive Health Enhancement Support System
IADL: instrumental activities of daily living
MCC: multiple chronic conditions
MDSPSS: Multidimensional Scale of Perceived Social Support
PSS: Perceived Stress Scale
RCT: randomized controlled trial
RMBPC: Revised Memory and Behavior Problems Checklist
RSCS: Revised Scale for Caregiving Self-Efficacy
STAR: Skills Training and Reskilling
ZBI: Zarit Burden Index
