# Supplementary material for: Web-Based Interventions to Improve Mental Health, General Caregiving Outcomes, and General Health for Informal Caregivers of Adults With Chronic Conditions Living in the Community: Rapid Evidence Review
Source: J Med Internet Res. 2017 Jul 28;19(7):e263. doi: 10.2196/jmir.7564 (PMC5554353; doi:10.2196/jmir.7564)
Supplement: Multimedia Appendix 1 [file jmir_v19i7e263_app1.pdf]

Multimedia Appendix 1. Search terms

Search Terms Ageline:

"((DE "Internet") OR (AB (internet OR web OR social media OR blog* OR social networking OR twitter* OR tweet* OR online OR instant* messag* OR text messag* OR text OR texts OR texting OR facebook* OR myspace OR linkedin OR email* OR e-mail* OR electronic mail OR app OR apps OR message board* OR electronic OR e-health OR telecommunication*)) OR (AB (webcast* OR podcast* OR streaming video* OR RSS OR really simple syndication OR youtube))) AND ((DE "Caregivers") OR (DE "Adult Children" OR DE "Spouses") OR (AB (carer* OR caretaker* OR care-giver* OR caregiver*)) OR (AB (adult child* OR spous*))) AND ((DE "Adults of All Ages") OR (AB (adult OR adults OR senior* OR elderly OR frail)) OR (DE "Dementia" OR DE "Alzheimers Disease" OR DE "Early Onset Dementia" OR DE "Frontotemporal Dementia" OR DE "Lewy Body Dementia" OR DE "Vascular Dementia") OR (AB (dementia* OR alzheimer*)))

Search Terms Cochrane:

#1 MeSH descriptor: [Internet] explode all trees
#2 MeSH descriptor: [Social Networking] this term only
#3 MeSH descriptor: [Electronic Mail] explode all trees
#4 MeSH descriptor: [Text Messaging] explode all trees
#5 MeSH descriptor: [Telecommunications] this term only
#6 (internet or web or social media or blog* or social networking or twitter* or tweet* or online or instant* messag* or text messag* or text or texts or texting or facebook* or myspace or linkedin or email* or e-mail* or electronic mail or app or apps or message board* or electronic or e-health or telecommunication*):ti,ab,kw (Word variations have been searched)
#7 MeSH descriptor: [Webcasts] this term only
#8 (webcast* or podcast* or streaming video* or RSS or really simple syndication or youtube):ti,ab,kw (Word variations have been searched)
#9 S #1 or S #2 or S #3 or S #4 or S #5 or S #6 or S #7 or S #8
#10 MeSH descriptor: [Caregivers] this term only
#11 MeSH descriptor: [Family] this term only
#12 MeSH descriptor: [Adult Children] this term only
#13 MeSH descriptor: [Spouses] this term only
#14 (carer* or caretaker* or care-giver* or caregiver*):ti,ab,kw (Word variations have been searched)
#15 (adult child* or spous*):ti,ab,kw (Word variations have been searched)
#16 s #10 or s #11 or s #12 or s #13 or S #14 or S #15
#17 MeSH descriptor: [Adult] explode all trees
#18 (adult or adults or senior* or elderly or frail):ti,ab,kw (Word variations have been searched)
#19 MeSH descriptor: [Dementia] explode all trees
#20 (dementia* or alzheimer*):ti,ab,kw (Word variations have been searched)
#21 s #17 or s #18 or S #19 or S #20
#22 s #9 and s #16 and s #21 Publication Year from 1995 to 2016

Search Terms Embase:

1. Internet/; 2. internet.ti,ab.; 3. web.ti,ab.; 4. social media/; 5. social media.ti,ab.; 6. blog*.ti,ab.; 7. (app or apps).ti,ab.; 8. twitter*.ti,ab.; 9. tweet*.ti,ab.; 10. online.ti,ab.; 11. message board*.ti,ab.; 12. instant* messag*.ti,ab.; 13. text messaging/; 14. text messag*.ti,ab.; 15. text*.ti,ab.; 16. facebook.ti,ab.; 17. myspace.ti,ab.; 18. linkedin.ti,ab.; 19. e-mail/; 20. email*.ti,ab.; 21. e-mail.ti,ab.; 22. webcast/; 23. electronic.ti,ab.; 24. social networking.ti,ab.; 25. (webcast* or podcast* or streaming video* or RSS or really simple syndication or youtube).ti,ab.; 26. or/1-25; 27. caregiver/; 28. (carer* or care giver* or caregiver* or caretaker*).ti,ab.; 29. family/; 30. adult child/; 31. spouse/; 32. (adult child* or spous*).ti,ab.; 33. or/27-32; 34. exp adult/; 35. pensioner/; 36. (adult or adults or middle aged or elderly or frail or pensioner*).ti,ab.; 37. exp Dementia/; 38. (dementia* or Alzheimer*).ti,ab.; 39. or/34-38; 40. 26 and 33 and 39 ; 41. limit 40 to (english language and yr="1995 -Current")

Search Terms Medline:

1. internet/ or blogging/ or social media/; 2. Social Networking/; 3. electronic mail/ or text messaging/; 4. Telecommunications/; 5. (internet or web or social media or blog* or social networking or twitter* or tweet* or online or instant* messag* or text messag* or text or texts or texting or facebook* or myspace or linkedin or email* or e-mail* or electronic mail or app or apps or message board* or electronic or e-health or telecommunication*).ti,ab.; 6. webcasts/; 7. (webcast* or podcast* or streaming video* or RSS or really simple syndication or youtube).ti,ab.; 8. or/1-7; 9. Caregivers/; 10. Family/ or adult children/; 11. Spouses/; 12. (carer* or caretaker* or care-giver* or caregiver*).ti,ab.; 13. (adult child* or spous*).ti,ab.; 14. or/9-13; 15. exp adult/; 16. (adult or adults or senior* or elderly or frail).ti,ab.; 17. exp Dementia/; 18. (dementia* or alzheimer*).ti,ab.; 19. or/15-18; 20. 8 and 14 and 19; 21. limit 20 to english language; 22. limit 21 to yr="1995 -Current"; 23. remove duplicates from 22

Search Terms PsychInfo:

1. internet/; 2. social media/ or electronic communication/ or online social networks/ or exp computer mediated communication/; 3. electronic communication/ or blog/ or text messaging/; 4. (internet or web or social media or blog* or social networking or twitter* or tweet* or online or instant* messag* or text messag* or text or texts or texting or facebook* or myspace or linkedin or email* or e-mail* or electronic mail or app or apps or message board* or electronic or e-health or telecommunication*).ti,ab.; 5. (webcast* or podcast* or streaming video* or RSS or really simple syndication or youtube).ti,ab.; 6. or/1-5; 7. caregivers/; 8. FAMILY/; 9. adult offspring/; 10. SPOUSES/; 11. (carer* or caretaker* or care-giver* or caregiver*).ti,ab.; 12. (adult child* or spous*).ti,ab.; 13. or/7-12; 14. (adult or adults or senior* or elderly or frail or middle aged).ti,ab.; 15. exp DEMENTIA/; 16. (dementia* or alzheimer*).ti,ab.; 17. or/14-16; 18. 6 and 13 and 17; 19. limit 18 to (english language and yr="1995 -Current")

Search Terms CINAHL:

S25 S11 AND S17 AND S22 Limiters - Published Date: 19950101-20161231; English Language
S24 S11 AND S17 AND S22 Limiters - Published Date: 19950101-20161231
S23 S11 AND S17 AND S22 Search modes - Boolean/Phrase Interface - EBSCOhost

Research Databases
S22 S18 OR S19 OR S20 OR S21 Search modes - Boolean/Phrase Interface - EBSCOhost Research Databases
S21 TI ((dementia* or alzheimer*)) OR AB ((dementia* or alzheimer*)) Search modes - Boolean/Phrase Interface - EBSCOhost Research Databases
S20 (MH "Dementia+") Search modes - Boolean/Phrase Interface - EBSCOhost Research Databases
S19 TI ((adult or adults or senior* or elderly or frail or pensioner* or middle aged)) OR AB ((adult or adults or senior* or elderly or frail or pensioner* or middle aged)) Search modes - Boolean/Phrase Interface - EBSCOhost Research Databases
S18 (MH "Adult+") Search modes - Boolean/Phrase Interface - EBSCOhost Research Databases
S17 S12 OR S13 OR S14 OR S15 OR S16 Search modes - Boolean/Phrase Interface - EBSCOhost Research Databases
S16 TI ((adult child* or spous*)) OR AB ((adult child* or spous*)) Search modes - Boolean/Phrase Interface - EBSCOhost Research Databases
S15 (MH "Spouses") Search modes - Boolean/Phrase Interface - EBSCOhost Research Databases
S14 (MH "Family") OR (MH "Adult Children") Search modes - Boolean/Phrase Interface - EBSCOhost Research Databases
S13 TI ((carer* or caretaker* or care-giver* or caregiver*)) OR AB ((carer* or caretaker* or care-giver* or caregiver*)) Search modes - Boolean/Phrase Interface - EBSCOhost Research Databases
S12 (MH "Caregivers") Search modes - Boolean/Phrase Interface - EBSCOhost Research Databases
S11 S1 OR S2 OR S3 OR S4 OR S5 OR S6 OR S7 OR S8 OR S9 OR S10 Search modes - Boolean/Phrase Interface - EBSCOhost Research Databases
S10 (MH "Social Networking") Search modes - Boolean/Phrase Interface - EBSCOhost Research Databases
S9 (MH "Webcasts+") Search modes - Boolean/Phrase Interface - EBSCOhost Research Databases
S8 TI ((webcast* or podcast* or streaming video* or RSS or really simple syndication or youtube)) OR AB ((webcast* or podcast* or streaming video* or RSS or really simple syndication or youtube)) Search modes - Boolean/Phrase Interface - EBSCOhost Research Databases
S7 TI ((internet or web or social media or blog* or social networking or twitter* or tweet* or online or instant* messag* or text messag* or text or texts or texting or facebook* or myspace or linkedin or email* or e-mail* or electronic mail or app or apps or message board* or electronic or e-health or telecommunication*)) OR AB ((internet or web or social media or blog* or social networking or twitter* or tweet* or online or instant* messag* or text messag* or text or texts or texting or facebook* or myspace or linkedin or email* or e-mail* or electronic mail or app or apps or message board* or electronic or e-health or telecommunication*)) Search modes - Boolean/Phrase Interface - EBSCOhost Research Databases
S6 (MH "Social Media") Search modes - Boolean/Phrase Interface - EBSCOhost Research Databases
S5 (MH "Electronic Mail") Search modes - Boolean/Phrase Interface - EBSCOhost Research

Databases
S4 (MH "Blogs") Search modes - Boolean/Phrase Interface - EBSCOhost Research Databases

S3 (MH "World Wide Web Applications") Search modes - Boolean/Phrase Interface - EBSCOhost Research Databases
S2 (MH "World Wide Web") Search modes - Boolean/Phrase Interface - EBSCOhost Research Databases
S1 (MH "Internet") Search modes - Boolean/Phrase Interface - EBSCOhost Research Databases

Note: Search developed by Laura Banfield and Jo-Anne Petropoulos, Health Sciences Library, McMaster University.
