# Supplementary material for: Web-Based Interventions to Improve Mental Health, General Caregiving Outcomes, and General Health for Informal Caregivers of Adults With Chronic Conditions Living in the Community: Rapid Evidence Review
Source: J Med Internet Res. 2017 Jul 28;19(7):e263. doi: 10.2196/jmir.7564 (PMC5554353; doi:10.2196/jmir.7564)
Supplement: Multimedia Appendix 2 [file jmir_v19i7e263_app2.pdf]

Multimedia Appendix 2. Characteristics of outcome studies

Author and Year<br>Country<br>Design	Study Population and Sample Size	Intervention Components and Description	Control	Measures and Time Point of Analyses	Outcomes
Beauchamp et al (2005) [38]<br>USA<br>RCT	299 employed family caregivers of persons with dementia<br>IG: n=150<br>CG: n=149<br>Age: 46.9 (12.2); female: 73%	**Information or Education**<br>Caregiver's Friend: Dealing with Dementia<br>Multicomponent intervention: knowledge, cognitive and behavioral skill, and affective learning presented across three modules: being a caregiver (common issues); coping with emotions; and common difficulties. Program provided text and videos that model positive caregiving strategies.	Control group allowed access to intervention after 30 day waitlist	**Anxiety**: State-Trait Anxiety Inventory<br>**Caregiver gain**: Positive Aspects of Caregiving survey<br>**Depressive symptoms**: Center for Epidemiological Studies Depression Scale (CES–D)<br>**Self-efficacy:** 6 questions<br>**Strain**: Caregiver Strain Instrument<br>**Stress**: 2 questions<br>**Ways of coping**: Revised Ways of Coping<br>Post test: 30 days	**Anxiety:**<br>IG Pre: 15.6(5.8);<br>Post: M 14.7 (5.6);<br>CG Pre: 14.7(6.4);<br>Post: 15.6(6.4),<br>***P***=.03<br><br>**Caregiver gain:**<br>IG Pre: 30.9 (7.0);<br>Post: 32.6 (7.1);<br>CG Pre: 30.8(6.7);<br>Post: 30.9(6.7), ***P***=.02<br><br>**Depressive symptoms:**<br>IG Pre: 16.8(11.3);<br>Post: 15.4(9.7);<br>CG Pre: 15.4 (10.9);<br>Post: 16.4(11.2),<br>***P***=.009<br><br>**Stress:**<br>IG Pre: 14.2 (7.7);<br>Post: 10.8 (6.2);<br>CG Pre: 12.3 (7.0);<br>Post: 11.6 (6.4),<br>***P***=<.001<br><br>**Self efficacy:**<br>IG Pre: 23.2 (7.9);<br>Post: 25.7(7.4);<br>CG Pre: 22.7(7.9);<br>Post: 23.5(8.1),<br>***P***=.02

					**Caregiver strain:**<br>IG Pre: 41.2(10.3);<br>Post: 39.1(9.5);<br>CG Pre: 39.8(9.7);<br>Post: 39.9(9.7),<br>***P***=.03<br><br>**Coping:**<br>IG Pre: 66.5 (11.1);<br>Post: 67.5 (11.6);<br>CG Pre: 65.3 (11.6);<br>Post: 66.3 (10.5),<br>***P***=.97
Blom et al (2015) [45]<br>Netherlands<br>RCT	245 family caregivers of people with dementia<br>IG: n=149;<br>age: 61.5 (11.9);<br>female: 70%<br>CG: n=96;<br>age: 60.8 (13.1);<br>female: 69%	**Information or Education + Psychosocial support - Professional**<br>Mastery over Dementia (MoD)<br>Internet course designed to reduced CG depression and anxiety; consists of 8 lessons and a booster session with the guidance of a coach monitoring the progress of participants and evaluating the homework. Each lesson consists of information (text material and videos), exercises and homework with evaluation at the start and end of each session	Minimal intervention consisting of e-bulletins (digital newsletters) with practical information on caring for person with dementia; no contact with a coach	**Anxiety**: Hospital Anxiety and Depression Scale (HADS-A)<br>**Depressive symptoms**: Center for Epidemiological Studies Depression Scale (CES-D 20)<br>Post-test: after the last session, 5-6 months after baseline	**Anxiety:**<br>IG Pre: 8.36; Post: 6.68, Mean change=1.69 SD=3.26;<br>CG Pre: 7.77; Post: 7.30, Mean Change=0.47 SD=3.41,<br>***P***=.008 (moderate effect size .48)<br>**Depressive symptoms:**<br>IG Pre: 17.89; Post: 15.55, Mean change=2.35 SD=8.21;<br>CG Pre: 16.61; Post: 16.95, Mean change -0.34 SD=7.51, ***P***=.02 (small effect size .26)
Cristancho-Lacroix et al (2015) [46]<br>France<br>RCT	49 caregivers of persons with dementia who spend at least 4 hours per week with relative,	**Information or Education + Psychosocial support - Peer**<br>Diapason<br>Multicomponent program combining information and	CG and IG received usual care, information about illness during semiannual follow-up with geriatrician.	**Burden**: Zarit Burden Interview (ZBI)<br>**Depressive symptoms**: Beck Depression	**Burden:**<br>IG Pre: 38.0 (14.5);<br>Post: 39.6 (15.7);<br>CG Pre: 35.0 (15.0);<br>Post: 34.8 (15.9),

	score 12 or more on Perceived Stress Scale<br>IG: n=25; Age: 64.2 (10.3); female: 64%<br>CG: n=24; Age: 59.0 (12.4); female: 67%	interaction between caregivers. Three month program with 12 weekly online sessions, sequentially and weekly unblocked once previous one was entirely viewed. Each session included theoretical and practical information, videos of health professionals and practice guide for applying the session's content. Website included sections on relaxation training, life stories about 4 couples where difficult situations are illustrated and possible solutions discussed; private and anonymous forum for caregivers to share with peers. Weekly sessions 15-30 minutes.		Inventory (BDI-II)<br>**Perceived stress**: Perceived Stress Scale (PSS-14)<br>**Reaction to problem behaviors:** Revised Memory and Behavior Problems Checklist (RMBPC) scale<br>**Self-efficacy**: Revised Scale for Caregiving Self-Efficacy (RSCS)<br>**Self-perceived health**: Nottingham Health Profile (NHP): **Social isolation; Emotions; Energy**<br>**Visual Analog Scale (VAS)** with 4 items: **Knowledge** (about Alzheimer's disease); **stress**; **coping** (with the illness); **quality of relationship** (between caregiver-person with dementia)<br>Post-test: 3 and 6 months (***P*** values reported for month 3-month 0 difference)	***P***=.74<br><br>**Depressive symptoms:**<br>IG Pre: 11.2(10.1); Post: 12.4 (11.6);<br>CG Pre: 9.0 (7.4); Post: 8.8 (7.2), ***P***=.56<br>**Perceived stress**:<br>IG Pre: 24.2 (9.0); Post: 25.0 (9.9);<br>CG Pre: 24.5 (6.7); Post: 23.8 (6.9), ***P***=.98<br><br>**Reaction to problem behaviors:**<br>IG Pre: 2.2 (0.4); Post: 2.3 (0.5)<br>CG Pre: 2.2 (0.6); Post: 2.1 (0.6), ***P***=.66<br><br>**Self-efficacy**:<br>IG Pre: 72.2 (17.0); Post: 71.5 (23.1);<br>CG Pre: 66.3 (18.2); Post: 68.4 (15.3), ***P***=.52<br>**Self-perceived health: Social isolation:**<br>IG Pre:14.1 (20.4); Post:16.5 (23.4);<br>CG Pre: 12.5 (17.2); Post: 14.8 (20.7), ***P***=.79;<br>**Emotions:**<br>IG Pre: 20.6 (22.4); Post: 26.6 (25.6);<br>CG Pre: 18.6 (20.3);

					Post: 17.2 (19.2), ***P***=.84;<br>**Energy:**<br>IG Pre: 27.9 (39.1);<br>Post: 35.9 (39.4);<br>CG Pre:26.6 (31.7);<br>Post:35.6 (41.6),<br>***P***=.22<br><br>**VAS: Knowledge:**<br>IG Pre: 45.4(23.2);<br>Post: 58.6 (24.4);<br>CG Pre: 44.5 (23.5);<br>Post: 51.7 (18.8),<br>***P***=.008<br>**Stress**:<br>IG Pre: 40.7 (23.0)<br>Post: 50.6 (23.2);<br>CG Pre: 50.2 (15.3);<br>Post: 50.3 (17.0),<br>***P***=.05<br>**Coping:**<br>IG Pre: 67.4 (15.8);<br>Post: 67.2 (17.6);<br>CG Pre: 61.4 (21.8);<br>Post: 61.8 (17.5),<br>***P***=.71<br>**Quality of relationship:**<br>IG Pre: 71.4 (20.5);<br>Post: 72.7 (17.9);<br>CG Pre: 72.1 (16.9);<br>Post: 69.3 (18.0),<br>***P***=.36
Dew et al (2004) [33]<br>USA (University of Pittsburgh Medical Center)<br>CCT	60 caregivers of heart transplant recipients<br>IG: n=20; age: ≤55 years 45%; female: 85%	**Information or Education + Psychosocial support - Peers +Psychosocial support - Professional**<br><u>HeartNet website:</u><br>Website included two interactive	Control group of people who did not have access to the website from other longitudinal studies who were matched on age,	**Mental health**: depression, anxiety and anger-hostility symptoms: subscales from Symptom Checklist-90 (SCL-90)	**Anxiety:**<br>F=3.91, ***P***=.05<br><br>**Depressive symptoms**: ***P***>.05

	CG: n=40; age: ≤55 years: 65%; female: 85%	stress and medical regimen management workshops, separate monitored discussion groups for patients and caregivers, access to electronic communication with transplant team, information on transplant-related health issues, resources and references library; 4 months of website use	education, income and relationship to the family caregiver	Post-test: 4-6 months	**Anger-hostility**:<br>F =4.90, ***P***=.03
DuBenske et al (2014)* [30]<br><br>USA<br><br>RCT	285 patient-caregiver dyads of persons with advanced nonsmall cell lung cancer<br><br>IG: n=144; age: 56.56 (12.86); female: 66.1%<br><br>CG: n=141; age: 54.57(12.21); female: 70.5%	**Information or Education + Psychosocial support - Peers + Psychosocial support - Professional**<br><br>CHESS: (Comprehensive Health Enhancement Support System) includes: (a) information services (eg, web links, resource directory, real-life text accounts of coping with cancer); (b) communication services (eg, discussion groups, ask an expert clinician reports); (c) coaching and training (eg, decision aids, action plan).	Usual care with internet access; list of high-quality patient-directed lung cancer and palliative care websites	**Burden**: Caregiver Quality of Life-Cancer Scale (CQOLC) Burden Subscale<br><br>**Disruptiveness**: Caregiver Quality of Life-Cancer Scale (CQOLC) Disruptiveness Subscale<br><br>**Negative mood**: Short Version Profile of Mood States (SV-POMS) (tension-anxiety, anger-hostility, depression-dejection)<br><br>Post-test: 6 months	**Burden:**<br>IG: M=12.97, SE=1.42;<br>CG: M=16.37 SE=1.43; ***P***=.02<br><br>**Disruptiveness:**<br>IG: M=3.17, SE=.95;<br>CG: M = 4.58, SE=.97; ***P***=.15<br><br>**Negative mood:**<br>IG: M=.56, SE=.13;<br>CG: M=.92, SE=.13;<br>***P***=.006
Hattink et al (2015) [47]<br>Netherlands and United Kingdom<br>RCT	72 informal caregivers of person with dementia living in the community and 24 volunteers in community dementia care<br>Completers:<br>IG: n=27; age Mean, (SD): 52.9 (11.4); female: 74%;<br>CG: n=32; age: 54.7	**Information or Education + Psychosocial Support - Peers**<br><br>STAR (Skills Training and Reskilling)<br>Multilingual e-learning tool provides dementia care training for both informal and formal caregivers<br>Online course with 8 modules on different topics in dementia care; modules consist of text, videos,	Randomly allocated to a 4-month waitlist for access to STAR	**Burden** (1 question) (informal caregivers only)<br>**Distress:** (subscale of Interpersonal Reactivity Index (IRI))<br>**Empathy**: Interpersonal Reactivity Index (IRI) (subscales include distress, empathy, fantasy, perspective)<br>**Knowledge on**	**Burden**:<br>IG Pre: 2.67 (1.11); Post:2.43 (0.98)<br>CG Pre: 3.08 (1.13); Post: 2.80 (0.96),<br>***P***=.43<br><br>**Distress**,<br>IG Pre:14.33 (6.20); Post: 9.74 (5.33);<br>CG Pre:14.25 (5.85); Post: 13.59 (5.63),

	(14.4); female: 69%	interactive exercises (quizzes), knowledge tests, references to other websites, literature, videos. Learning Path Advisor in STAR that assesses baseline knowledge and confidence to help people decide which module to start with. Facebook communities to promote peer support for all nationalities of users.		**dementia and attitudes regarding dementia:** Alzheimer's Disease Knowledge Scale (ADKS); Approaches to Dementia Questionnaire (ADQ)<br>**Perspective:** (subscale of Interpersonal Reactivity Index (IRI))<br>**Quality of Life** (2 questions) (informal caregivers only)<br>**Sense of Competence**: Short Sense of Competence Questionnaire (Informal caregivers only)<br>Post-test: 2-4 months after baseline	***P***=.003<br><br>**Empathy**:<br>IG Pre:12.56; (6.45);<br>Post: 20.40 (4.06);<br>CG Pre: 12.81 (6.60);<br>Post: 13.03 (5.63),<br>***P***<.001<br>**Knowledge:**<br>IG Pre: 69.15 (6.74);<br>Post: 71.59 (6.48);<br>CG Pre: 60.13 (10.4):<br>Post: 64.66 (4.90)<br>***P***=.001<br>**Attitudes:**<br>IG Pre: 24.67 (3.43);<br>Post: 24.44 (3.11);<br>CG Pre: 24.13 (3.32);<br>Post: 24.28 (3.12);<br>***P***=.90<br>**Perspective**:<br>IG Pre:13.11 (5.66);<br>Post: 18.81(3.45);<br>CG Pre: 13.06 (5.79)<br>Post: 13.75(4.45),<br>***P***<.001<br>**Quality of life:**<br>IG Pre: 7.24 (1.58);<br>Post: 7.05 (1.77)<br>CG Pre: 6.23 (1.75);<br>Post: 6.48 (1.58),<br>***P***=.97<br><br>**Sense of competence:**<br>IG Pre: 4.43 (1.25);<br>Post: IG=4.67 (1.06);<br>CG Pre: 4.54 (1.56)<br>Post: 4.04 (1.49),<br>***P***=.02

Hattink et al (2016) [37]<br>Germany (RCT), Netherlands and Belgium (CCT)	32 carers of persons with mild cognitive impairment or dementia living in the community<br>IG: n=17; Age: 57.8 (3.1);<br>CG: n=15; Age 61.7 (5.0);<br>Female: 50%	**Monitoring + Psychosocial support – Peers + Psychosocial support - Professional**<br>Rosetta<br>System included: *Home Navigation:* Elderly Day Navigator; video home terminal or mobile device, provides reminders for activities; phone with photo address book; to promote safety, warnings on screen and help button to enable direct phone contact with relative; GPS technology on mobile phone so caregiver could see on webpage where person is and guide them home; *Surveillance:* Early Detection System software; recording of daily patterns of behavior; warning to carers if changes in day to day pattern, creation of graphs to help informal and professional carers decide on need for followup; *Monitoring:* Advanced Awareness and Prevention System. Generates alarms to care organizations of emergencies, eg, falls, using speak-listen option and camera feed. Used for 2 weeks to 8 months; could choose the functions wanted.	Received usual care and support, including home care for assistance with household chores, or personal and day care.	**Quality of life**: Quality of Life in Alzheimer's Disease Scale (QoL-AD)<br>**Sense of competence**: Short Sense of Competence Questionnaire (SSCQ)<br>Post-test: 4 months	**Quality of life:**<br>IG Pre: 31.89 (2.05); Post: 30.25 (6.74);<br>CG Pre: 29.00 (0.96) Post: 30.13 (3.87), ***P***=.37<br><br>**Sense of competence:**<br>IG Pre: 4.13 (0.40); Post: 4.13 (1.45)<br>CG Pre: 5.30 (0.37) Post: 5.13 (0.83), ***P***=.11

Kajiyama et al (2013) [39]<br>USA<br>RCT	150 caregivers of persons with dementia<br>Completers: IG: n=46<br>CG: n=57<br>Age: mean 56.1 (11.97)<br>Female: 84%	**Information or Education**<br>iCare Stress Management e-Training program<br>An internet based psychoeducational program aimed to teach set of core coping skills. 8 modules, completed in order, no minimum time for each; Participants encouraged to practice assignments in each module for 7-10 days before moving on; embedded video clips illustrating how to do skills presented; 3 month program	Education or information only	**Depressive symptoms:** Center for Epidemiological Studies Depression scale (CES-D 20)<br>**Quality of life:** Perceived Quality of Life (PQoL)<br>**Stress**: Perceived Stress Scale (PSS)<br>**Reaction to problem behaviors**: Revised Memory and Behavior Problems Checklist (RMBPC)<br>Post-test: 3 months	**Depressive symptoms:**<br>IG Pre: 16.71 (7.95); Post: 14.19 (7.68);<br>CG Pre: 14.10 (7.82); Post: 13.33 (9.31), ***P***=.26<br><br>**Quality of life:**<br>IG Pre: 5.85 (1.84); Post: 6.34 (1.54);<br>CG Pre: 6.25 (1.90); Post: 6.31 (1.84), ***P***=.12<br><br>**Stress:**<br>IG Pre: 18.46(5.20); Post: 15.83 (5.07);<br>CG Pre: 16.22 (6.87); Post: 16.41 (7.15), ***P***=.02<br><br>**Reaction to problem behaviors:**<br>IG Pre: 1.17 (0.69); Post: 0.83 (0.63);<br>CG Pre: 1.03 (0.66); Post: 0.91 (0.75), ***P***=.60
Klemm et al (2014) [36]<br>USA (University of Delaware employees)<br>CCT	86 employed family caregivers aged 40 years or older of persons with chronic illness (eg, dementia, diabetes, stroke, cancer, heart disease)<br>IG#1: Professionally facilitated n=20;<br>IG#2 moderated	**Information or Education + Psychosocial support - Peers + Psychosocial support - Professional**<br>Online support groups (OSG)<br>Two online support groups, IG#1: professionally facilitated psychoeducational, IG#2: moderated peer directed.<br>*IG#1:* facilitated by clinical nurse specialist in psychiatric	Nonactive (assigned to one of the OSGs) did not participate in online discussion; posted or read fewer than 4 messages over 12 weeks.	**Depressive symptoms**: Center for Epidemiological Studies Depression scale (CES-D 20)<br>**Caregiver Strain**: Modified Caregiver Strain Index (CSI)<br>**Quality of life**: Caregiver Qualify of Life Index (CQoL-I)	**Depressive symptoms:**<br>IG#1 vs CG ***P***=.04<br>IG#2 vs CG ***P***=.03<br>IG#1 vs IG#2 ***P***=.52<br><br>**Caregiver strain:**<br>IG#1 vs CG ***P***=.05<br>IG#2 vs CG ***P***=.10<br>IG#1 vs IG#2 ***P***=.37

	peer directed n=27;<br><br>CG n=39<br>Age: 52.2<br>Female: 91%	nursing in semi-structured format for 12 weeks using asynchronous communication<br>*IG#2:* moderated peer directed group monitored by researcher in unstructured format for 12 weeks using asynchronous communication		Post-test: 12 weeks	**Quality of life:**<br>IG#1 vs CG ***P***=.01<br>IG#2 vs CG ***P***=.008<br>IG#1 vs IG#2 ***P***=.71
Mahoney et al (2008) [35]<br>USA<br>CCT	27 family caregivers employed by one of 5 companies, caregiver of older adult who lived at home during the day, with at least one elder health or safety concern<br>Completers:<br>IG#1: Low Tech n=12; Female: 100%<br><br><br>IG#2: High Tech; n=7; Female: 57%<br>Age: 30s: 7%; 40s: 45%; 50s: 44%; 60s: 4%	**Information or Education + Monitoring + Psychosocial support - Professional**<br>Worker Interactive Networking (WIN) project<br>Workplace-based online caregiver support and remote monitoring of elders at home<br>*IG#1:* Low tech: online support group moderated by geriatric/psychiatric nurse that offered bulletin board discussion group with live chat room; moderated email for private interchanges and web page link to Alzheimer's Association staff for memory loss issues, direct web page link to geriatrician who offered free email consultations<br>*IG#2:* High Tech web based remote home monitoring of older adult in their homes using wireless sensors, accessible to caregiver, or with email or pager alert; 6 month program	No control	**Stress**: included several standardized indicators including caregiver vigilance, preparedness for caregiving<br>**Worker morale**: 5-item scale originated from Business Work-Life Study<br>**Productivity**: work time and quality of work reduced due to caregiving, originated from Business Work-Life Study<br>Post-test: 6 months	Items on some scales showed improvement but no power for statistical testing<br>**Stress: preparedness for stress of caregiving:**<br>IG#1: Pre: 1.64; Post: 2.00;<br>IG#2: Pre: 2.40; Post: 2.60

Marziali et al (2006) [44]<br>Canada<br>RCT	66 family caregivers of persons with Alzheimer's disease, stroke-related dementia and Parkinson's disease<br>Completers (n=38)<br>IG: n=23<br>CG: n=15<br>Age: 67.8; female: 76%	**Information or Education + Psychosocial support - Peers + Psychosocial support - Professional**<br>Caring for Others<br>Internet based video-conferencing psychosocial educational sessions. Group therapist met with 6 caregivers using Caring for Others video conferencing link for 1 hour per week x 10 weeks. Then a group member assumed the task of guiding weekly group sessions for an additional 12 weeks. Link to relevant educational materials Technicians installed equipment in caregiver homes and provided two computer training sessions.	Control (no Intervention)	**Depressive symptoms**: Center for Epidemiological Studies Depression scale (CES-D)<br>**Health-related quality of life**: Health Status Questionnaire 12<br>**Stress**: stress experienced in relation to performing activities of daily living (ADLs) and instrumental ADLs (IADLs) for care recipient<br>**Reaction to problem behaviors:** Revised Memory and Behavior Problems Checklist (RMBPC)<br>**Perceived social support**: Multidimensional Scale of Perceived Social Support<br>**Combination of two stress measures** (stress and reaction above)<br>Post-test 6 months	**Depressive symptoms:** NS<br><br>**Health-related quality of life:** NS<br><br>**Stress:** NS<br><br>**Reaction to problem behaviors:** NS<br><br>**Perceived social support:** NS<br><br>**Combination of two stress measures (ADL and IADL) and managing difficult behaviors:**<br>IG: Mean stress change score -1.326; CG Mean stress change score 2.519, ***P***<.004
Marziali et al (2011) [34]<br>Canada<br>CCT	91 caregivers of persons with dementia in three cities<br><br>IG#1: Online Chat Group n=40<br><br>IG#2 Online Video Conferencing Support Group n=51	**Information or Education + Psychosocial support - Peers +Psychosocial support - Professional**<br><br>IG# 1 – Online Chat Group<br>IG #2 – Online Video<br><br>Caring for Me<br>Two interventions compared:<br>*IG#1: Online Chat Group*	No control group	**Depressive symptoms**: Center for Epidemiologic Studies Depression Scale (CES-D)<br><br>**Distress**: Functional Autonomy Measurement System (SMAF) modified to assess caregiver distress re to 5	**Depressive symptoms:** NS<br><br>**Distress:**<br>IG#2 showed lower distress scores associated with managing care recipient's deterioration in mental function:

	Age: 65.51; female: 72%.	Access to information handbook and 6 videos on caregiving; participants introduced to chat group by clinician moderator who encouraged sharing of experiences and problem solving approaches; moderator visited chat group once per month to encourage participation. Access to website for 6 months.<br>*IG#2: Online Video Conferencing Support Group:* Online video conferencing psychotherapeutic support group facilitated by clinician plus access to caregiver information handbook; access to all site features except educational videos; each group (n=6) met weekly online for 1 hour for 20 weeks (10 facilitated weeks + 10 non-facilitated weeks); facilitator was health professional (2 nurses and 1 social worker).		functional areas of care recipient: mobility, communication, mental function, ADL, IADL<br><br>**General health**: Health Status Questionnaire (HSQ 12) (includes mental health subscale)<br><br>**Self-Efficacy:** Revised Scale for Caregiver Self-Efficacy<br><br>**Social support**: Multidimensional Scale of Perceived Social Support<br><br>Post-test: 6 months	IG#2: Pre: 1.61 (0.89); Post: 1.43 (0.82);<br>IG#1: Pre: 1.61 (0.89); Post: 1.58 (0.94); F=5.65, ***P***≤.02<br><br>**Distress:**<br>IG#1 showed lower distress scores associated with managing IADL:<br>IG#1: Pre: 1.22 (1.0); Post: 0.74 (0.56);<br>IG#2 Pre: .78 (0.67); Post: .76 (0.74); F=5.79, ***P***≤ .02<br><br>**General health: HSQ mental health:**<br>IG#2 greater improvement in mental health<br>IG#2:Pre: 60.84 (22.19); Post: 64.67 (19.88);<br>IG#1: Pre: 59.92 (23.38); Post: 57.10 (22.01), F=5.69, ***P***≤.02<br><br>**Self-efficacy**<br>IG#1 Pre: 69.89 (17.45);<br>Post: 74.55 (19.10);<br>IG#2 Pre: 69.52 (16.24);<br>Post: 75.69 (15.97); F=4.3, ***P***≤.04

					**Social support**: NS
McLaughlin et al (2013) [40]<br><br>USA<br><br>RCT	201 caregivers aged 18 years + of persons with traumatic brain injury<br><br>IG: n=104; female: 86.4%<br><br>CG: n=97; female: 88.4%	**Information or Education**<br><br><u>The Brain Injury Partners (BIP) Program</u><br>Site was developed to (a) train caregivers in advocacy skills with focus on effective communication; (*b*) help users find a broad range of services and supports through external links and library of articles; (*c*) provide strategies for stress reduction such as coping with guilt and burnout; and (*d*) help determine necessary supports such as independent living needs and transition planning. The training uses text, interactive video examples, and video-based skills exercises.	Access to Brain Injury Association of America website with information about managing stress, requesting help from friends and family, and obtaining services; main advocacy focus is legislative. Participants asked to use site for minimum of 30 minutes.	**Advocacy: Knowledge:** 18 questions<br>**Advocacy: Intentions**: 17 video situation tests<br>**Advocacy: Skill Application**<br>17 video situation tests<br><br>**Satisfaction with life**: Satisfaction with Life Scale (SLS)<br><br>Post-test: 3 months	**Knowledge:**<br>IG Pre: 0.59 (0.11)<br>Post: 0.66 (0.11)<br>CG Pre: 0.59 (0.11)<br>Post: 0.62 (0.11)<br>***P***=.03<br><br>**Advocacy: Intention**:<br>IG Pre: 3.40 (0.61)<br>Post: 3.86 (0.59)<br>CG Pre: 3.52 (0.56)<br>Post: 3.53 (0.64)<br>***P***<.001<br><br>**Advocacy: Skill:**<br>IG Pre: 3.52 (0.55)<br>Post: 3.97 (0.51)<br>CG Pre: 3.57 (0.56)<br>Post: 3.62 (0.58)<br>***P***<.001<br><br>**Satisfaction with life:**<br>IG Pre: 3.29 (1.63);<br>Post: 3.16 (1.81);<br>CG Pre: 3.27 (1.60);<br>Post: 3.47 (1.57),<br>***P***=.05; effect size .29
Namkoong et al (2012)* [31]<br><br>USA<br><br>Secondary analysis of RCT. See DuBenske et al (2014)	285 patient-caregiver dyads of persons with advanced nonsmall cell lung cancer<br><br>IG: n=141<br>CG: n=144<br><br>Age: 55.56 (12.55)	**Information or Education + Psychosocial support - Peers + Psychosocial support - Professional**<br><br><u>CHESS: (</u>Comprehensive Health Enhancement Support System) includes: (a) information services (eg, web links, resource directory, real-life text accounts	Usual care with internet access; list of high-quality patient-directed lung cancer and palliative care websites	**Bonding** – 5-item bonding scale (captures concepts of universality, group cohesiveness, informational and emotional support)<br><br>**Coping**: items from Brief Cope: active behavior, positive	**Bonding**:<br>IG Pre: 1.42 (0.98);<br>Post: 1.44 (0.84);<br>CG Pre: 1.19 (1.01);<br>Post: 1.08 (0.88);<br>***P***=.04<br><br>**Coping**:<br>**Active coping:**<br>IG Pre:1.92 (0.94);

	Female: 68.3%<br><br>Completers: (n=104 caregivers)	of coping with cancer); (b) communication services (eg, discussion groups, ask an expert clinician reports); (c) coaching and training (eg, decision aids, action plan).		reframing, instrumental support<br><br>Post-test: 6 months	Post: 1.50 (0.91);<br>CG Pre: 1.76 (1.07);<br>Post:1.36 (0.89),<br>***P***=.85<br>**Positive reframing:**<br>IG Pre: 1.78 (1.04);<br>Post: 1.25 (0.98);<br>CG Pre: 1.45 (1.01);<br>Post: 1.38 (1.09),<br>***P***=.06<br>**Instrumental support:**<br>IG Pre: 1.36 (0.96);<br>Post: 1.09 (0.98);<br>CG Pre: 1.26 (1.00);<br>Post: 1.06 (0.91),<br>***P***=.58
Pagan-Ortiz et al (2014) [12]<br><br>Puerto Rico and Massachusetts and Mexico<br><br>CCT	72 Spanish-speaking caregivers of persons with dementia, aged 42-78<br><br>Completers:<br>Pretest IG (n=17)<br>Posttest IG (n=15)<br><br>Pretest CG (n=23)<br>Posttest CG (n=17)	**Information or Education + Psychosocial support - Peers +Psychosocial support - Professional**<br><br><u>Cuidate Cuidador (website)</u><br>The site offers culturally competent information about ADRD in Spanish and English, practical instructions on managing dementia related behaviors, real stories from caregivers, information on self-care, comment section where caregivers can post and interact with other caregivers, and ask an expert resource section with information on resources and videos. IG attended four sessions of 1 – 1.5 hours each.	Participants received printed Spanish-language educational materials on Alzheimer's caregiving.	**Burden**: Zarit Burden Inventory<br><br>**Depressive symptoms**: Center for Epidemiological Studies Depression Scale (CES-D)<br><br>**Perceived mastery and competence**: Personal Mastery Scale (PMS)<br><br>**Perceived social support**: Lubben Social Network Scale (LSNS)<br><br>Post-test: 1 month	**Burden:**<br>IG Pre: 1.64 (0.61);<br>Post: 1.73 (0.81);<br>CG Pre: 1.78 (0.63);<br>Post: 1.66 (0.62),<br>***P***=.77<br><br>**Depression:**<br>IG Pre: 0.74 (0.52);<br>Post: 0.76 (0.60);<br>CG Pre: 0.88 (0.49);<br>Post: 0.78 (0.50),<br>***P***=.93<br><br>**Perceived mastery:**<br>IG Pre: 2.16 (0.53);<br>Post: 2.24 (0.41);<br>CG Pre: 2.08 (0.64);<br>Post: 2.02 (0.48),<br>***P***=.17<br><br>**Perceived social**

					**support:**<br>IG Pre: 2.96 (0.67);<br>Post: 2.91 (0.53);<br>CG Pre: 2.95 (1.09);<br>Post: 2.91 (0.73),<br>***P***=.98
Pierce et al (2009) [41]<br>Midwestern USA<br>RCT	73 carers for first-time stroke survivors who were novice Internet users<br>IG: n=36; Age: 54 (12.2); female: 69%<br>CG: n=37; Age: 55 (13.1); female: 81%	**Information or Education + Psychosocial support - Peers +Psychosocial support - Professional**<br>Caring~Web<br>Web-based stroke intervention with four components offered for one year: (a) linked web sites about stroke and caring; (b) customized educational information or tips specific to carers' needs; (c) email forum to ask a nurse specialist and a rehabilitation team (therapists, pharmacist, dietitian, social worker and physician) questions in private; and (d) non-structured email discussion amongst all participants facilitated by the nurse	Non-web users	**Depressive symptoms**: Center for Epidemiological Studies Depression Scale (CES-D)<br>**Satisfaction with life**: Satisfaction with Life Scale (SWLS) -<br>Post-test: 1 year	**Depressive symptoms:**<br>IG Pre: 12 (9.9); Post: 12.3 (9.8);<br>CG Pre: 11.3 (8.4);<br>Post 9.0 (9.1) ***P***=.48<br><br>**Satisfaction with life:**<br>IG Pre: 22.3 (7.1);<br>Post: 21.7 (6.3);<br>CG Pre: 24.1 (5.9);<br>Post: 24.6 (6.0),<br>***P***=.90
Smith et al (2012) [42]<br><br>16 States in USA<br><br>RCT	38 dyads of spouse caregivers and male stroke survivors<br>Completers n = 32<br>IG: n=15; Age: 55.3 (6.9)<br>CG: n=17; Age: 54.9 (12.9)	**Information or Education + Psychosocial support - Peers + Psychosocial support - Professional**<br>Adaptation of Caring~Web<br>Five components of intervention: (a) professional guide (PhD nursing student) who facilitated weekly topics in educational modules and chat room, used email messages to tailor support to CGs; (b) 11 educational videos depicting support group of	Access to resource room only; at outset, watched online video in which the same professional guide explained the features of the Resource Room and encouraged CGs to use it as a caregiving resource. A weekly caregiving tip was presented online, but none overlapped with content covered in	**Depressive symptoms**: Center for Epidemiological Studies Depression scale (CES-D)<br>**Mastery**: Mastery Scale<br>**Self-esteem**: Self-esteem Scale<br>**Social support**: MOS Social Support Survey<br>Post-test: 11 weeks	**Depressive symptoms:**<br>IG Pre: 13.9 (2.0);<br>Post: 13.4 (1.6);<br>CG Pre: 19.7 (1.8);<br>Post: 16.6 (1.5),<br>***P***<.01<br>**Mastery:** NS<br>IG Pre: 24.2 (0.7);<br>Post: 24.1 (0.5);<br>CG Pre: 23.6 (0.6);<br>Post: 24.4 (0.5)<br>**Self-esteem:** NS

		women discussing and illustrating stroke caregiving topics; (c) 2 online chat sessions weekly led by professional guide for 17 sessions, groups of 4-5; (d) private email and message board; (e) resource room including virtual online library with information on stroke, caregiving, link to other websites, quizzes, self assessment instruments, instructional videos.	the intervention condition.		IG Pre: 31.6 (1.7);<br>Post: 31.1 (0.7);<br>CG Pre: 31.9 (0.6);<br>Post: 32.6 (0.7)<br>**Social support:** NS<br>IG Pre: 37.0 (1.7);<br>Post: 33.8 (1.6);<br>CG Pre: 37.0 (1.6);<br>Post: 36.3 (1.5)
Steiner et al (2008) [43]<br>Ohio and Michigan, USA<br>Secondary analysis of Pierce et al (2009) RCT	73 caregivers of stroke survivors<br>IG: n=36; Age: 54 (12.2); female: 69%<br>CG: n=37; Age: 55 (13.1); female: 81%	**Information or Education + Psychosocial support - Peers +Psychosocial support - Professional**<br>Caring~Web<br>Web-based stroke intervention with four components offered for one year: (a) linked web sites about stroke and caring; (b) customized educational information or tips specific to carers' needs; (c) email forum to ask a nurse specialist and a rehabilitation team (therapists, pharmacist, dietitian, social worker and physician) questions in private; and (d) non-structured email discussion amongst all participants facilitated by the nurse	Non-web users	**Caregiver health**: self rated health item from Multidimensional Functional Assessment of Older Adults<br>**Emotional support** from family and friends (single item)<br>**Physical help** from family and friends (single item)<br>Post-test: 12 months	**Health:** NS<br>**Emotional support:** NS<br>**Physical help:** NS
Torkamani et al (2014) [48]<br><br>UK, Spain and Greece	60 caregivers of persons with dementia from 3 sites (n=20 per site)<br><br>IG: n=30; age: 57.57 (12.5); female: 55%	**Information or Education + Psychosocial support - Peers + Psychosocial support - Professional**<br>ALADDIN<br>Contains four key features: (a) ALADDIN TV: information and	Usual care	**Burden**: Zarit Burden Interview<br><br>**Depressive symptoms**: Beck Depression Inventory and Zung Depression Self Rating	**Burden:**<br>IG Pre: 36.6 (17.5);<br>Post: 39.74 (23.12);<br>CG Pre: 29.07 (9.67);<br>Post: 30.60 (17.64),<br>***P***=.19

RCT	CG: n=30; age: 63.93 (14.74); female: 45%	educational material, relaxation; (b) SOCIAL NETWORKING: forum for carers to communicate with each other; (c) MY TASKS: carers complete questionnaires about their own and relatives health that can generate clinical alerts, monitored by clinicians who can respond quickly; (d) CONTACT US: carer can alert clinical site and request contact.		Scale<br><br>**Caregiver distress**: Neuropsychiatric Inventory (NPI)<br><br>**Quality of life**: EuroQoL (EQ5D) (1 site); and Quality of Life Scale (QOLS) (2 sites)<br><br>Post-test: 3 and 6 months (***P*** values for 6 months)	**Depressive symptoms**: Not evaluated due to missing data<br><br>**Distress**:<br>IG Pre: 7.20 (6.10); Post: 6.82 (4.33); CG Pre: 3.85 (4.77); Post: 3.20 (2.34), ***P***=0.006<br>ANCOVA: ***P***>.05<br><br>**Quality of Life EQ5D:**<br>IG Pre: 0.65 (0.26); Post: 0.80 (0.06); CG Pre: 0.72 (0.23); Post: 0.62 (0.23), ***P***=.045<br>ANCOVA ***P*=**.027<br>**QOLS:**<br>IG Pre: 59.83 (13.58); Post: 75.28 (8.54); CG Pre: 67.30 (11.65); Post: 77.80 (12.01), ***P***=.56

Notes: IG: Intervention Group; CG: Control Group; NS: not significant; the data reported represent the last (longest) available data collection point per study outcome. *See Gustafson et al. (2013) [32] for methodological details.
