# Supplementary material for: Web-Based Interventions to Improve Mental Health, General Caregiving Outcomes, and General Health for Informal Caregivers of Adults With Chronic Conditions Living in the Community: Rapid Evidence Review
Source: J Med Internet Res. 2017 Jul 28;19(7):e263. doi: 10.2196/jmir.7564 (PMC5554353; doi:10.2196/jmir.7564)
Supplement: Multimedia Appendix 3 [file jmir_v19i7e263_app3.pdf]

Multimedia Appendix 3. Outcome measures

Outcome Measured	Outcome Measure or Scale	Used in Study
Anxiety	Hospital Anxiety and Depression Scale (HADS)	[45]
	State-Trait Anxiety Inventory (STAI)	[38]
Burden	Zarit Burden Interview (ZBI)	[12], [46], [48]
Caregiver Gain	Positive Aspects of Caregiving Survey (PAoCS)	[38]
Caregiver Strain	Caregiver Strain Index (CSI)	[36], [38]
Coping	Brief Cope Scale (BCS)	[31]
	Revised Ways of Coping (R-WoC)	[38]
Competence or Perspective	Short Sense of Competence Questionnaire (SSCQ)	[37], [47]
Depression	Beck Depression Inventory (BDI)	[46], [48]
	Center for Epidemiological Studies Depression Scale (CESD)	[36], [38], [39], [44], [45] [12], [34], [41], [42]
	Zung Depression Self Rating Scale (ZDSR)	[48]
	Symptom Checklist-90 (SCL-90)	[33]
Disruptiveness, Burden	Caregiver Quality of Life - Cancer Subscale (CQoLC)	[30]
Distress, Empathy, Knowledge, Attitudes	Interpersonal Reactivity Index (IRI)	[47]
Knowledge	Alzheimer's Disease Knowledge Scale (ADSK)	[47]
Knowledge, Self-efficacy, Quality of relationship	Visual Analog Scale (VAS)	[46]
Life Satisfaction	Satisfaction with Life Scale (SLS)	[40], [41]
Mastery	Mastery Scale (MS)	[42]
	Personal Mastery Scale (PMS)	[12]
Negative Mood	Short Version Profile of Mood States (SV-POMS)	[30]
Overall Health	Multidimensional Functional (MFAOA)	[43]
	Nottingham Health Profile (NHP)	[46]
Quality of Life	Caregiver Quality of Life Index (CQoL-I)	[36]
	EuroQoL (EQ5D)	[48]
	Perceived Quality of Life (PQoL)	[39]
	Quality of Life in Alzheimer's Disease Scale (QoLAD)	[37]
	Quality of Life Scale (QoLS)	[48]

Quality of Life, Overall Health	Health Status Questionnaire (HSQ)	[34], [44]
Reaction to Problem Behavior	Revised Memory and Problem Behavior Checklist (RMPBC)	[39], [44], [46]
Self-Efficacy	Revised Scale for Caregiving Self-Efficacy (RSCS)	[34], [46]
Self-Esteem	Self-Esteem Scale (SES)	[42]
Social Support	Lubben Social Network Scale Assessment of Older Adults (LSNS)	[12]
	Multidimensional Scale of Perceived Social Support (MSPSS)	[34], [44]
	Social Support Survey (SSS)	[42]
Stress or Distress	Neuropsychiatric Inventory (NPI)	[48]
	Perceived Stress Scale (PSS)	[39], [46]
	Modified Functional Autonomy Measurement System (SMAF)	[34]
