# Supplementary material for: Web-Based Interventions to Improve Mental Health, General Caregiving Outcomes, and General Health for Informal Caregivers of Adults With Chronic Conditions Living in the Community: Rapid Evidence Review
Source: J Med Internet Res. 2017 Jul 28;19(7):e263. doi: 10.2196/jmir.7564 (PMC5554353; doi:10.2196/jmir.7564)
Supplement: Multimedia Appendix 4 [file jmir_v19i7e263_app4.pdf]

Multimedia Appendix 4. Website use and influence on outcomes

Author and Intervention	Website Use and Influence on Outcomes
Beauchamp et al (2005) [38]	**Use**: 59% visited once; 41% visited more than once (19% twice, 11% three times, and 11% four or more times). Mean total time spent across all visits: 32.2 min (SD=43.5; range=1–368).<br><br>**Influence of Use on Outcomes**: Authors created a composite outcome measure by calculating pretest-post test gain scores for each of the eight outcome measures (eg, depressive symptoms, anxiety, strain, stress, coping), standardized each gain score and computed mean value of standardized gain score. The correlation between time spent in the program and the composite gain score was significant at r=.30, ***P***<.001; more time spent was associated with significantly greater change in outcomes.
Blom et al (2015) [45]	No data provided regarding use.
Cristancho-Lacroix et al (2015) [46]	**Use**: 71% viewed at least 10 of 12 online sessions; average use of site: 19.72 times (SD=12.88); connected for 262.20 min (SD=270.74) during the first 3 months. After month 3, connection times near zero.<br><br>**Influence of Use on Outcome**: no significant correlation between perceived stress score and frequency (***P***=–.15) or duration (***P***=–.05) of use of the website.
Dew et al (2004) [33]	**Use**: Monthly or less: 41.2%; several times a month: 35.3%; weekly or more often: 23.5%. Average duration of use on each occasion:<br>15 min: 35.3%; 15 to 30 min: 52.9%; ≥30 min: 11.8%.<br><br>**Influence of Use on Outcomes**: greater website use was significantly correlated with greater improvement on role functioning related to emotional factors on the quality of life domain (r=0.49, ***P***=.049).
DuBenske et al (2014) [30]	**Use**: 73% logged on at least once during the first 6 months; average use during 6 months was 14.6 logins (*SD*=25.1), 293.0 pages (*SD*=482.1), and 177.7 min (*SD*=245.0). Average of 50.0 min per month (*SD*=42.9).
Hattink et al (2015) [47]	No data provided regarding use.
Hattink et al (2016) [37]	**Use**: Average use was nearly 4 months (range 2 weeks to 8 months); reasons for relatively short average period of usage: dropouts before installation of system; time to plan installation; technical issues.
Kajiyama et al (2013) [39]	**Use**: average use of 6.42 times per month.<br><br>**Influence of Use on Outcomes:** Website usage and helpfulness measures were not significantly correlated with change in any outcome measures.
Klemm et al (2014)	**Use**: Active participation described as those who read or posted more

[36]	than 4 messages over 12 weeks. Mean numbers of messages read by active participants in the professionally facilitated psychoeducational groups were 99.85 (range, 8–221) and 156.25 (range, 5–360) for the moderated peer directed groups. The mean numbers of messages posted by the professionally facilitated psychoeducational groups were 5.5 (range, 0–20) and 9.5 (range, 0–34) for the moderated peer directed groups.
Mahoney et al (2008) [35]	**Use:** total of 2,001 website "actions" occurred. Those in the "high-tech" (web-based remote monitoring [HM]) groups accounted for the majority of site activity. A core group of heavy users used the system features several times a day in contrast to others with more intermittent daily, weekly, or passive usage.
Marziali & Donahue (2006) [44]	No data provided regarding use.
Marziali & Garcia (2011) [34]	No data provided regarding use.
McLaughlin et al (2013) [40]	**Use**: 51% made 1 visit to the website, 24% made 2 visits, 18% made 3 or more visits, and 7% did not visit the website. The average time spent on the website across all visits was 57 min (SD=47 min), with a median of 45 min.<br><br>**Influence of Use on Outcomes:** significant correlation between more time on the website and change in knowledge (r=0.24, ***P***=.02).
Namkoong et al (2012) [31]	Secondary analysis of DuBenske et al.
Pagan-Ortiz et al (2014) [12]	**Use**: Most participants visited the website at least 3 times; others reported visiting the site at least 10 times or every other day. The average visit time lasted between 30 min and 1 hr.
Pierce et al (2009) [41]	**Use**: average use of website: 1–2 h per week. During the study, there were 7121 'hits' on the website. There were 102 messages to the nurse specialist in the email forum and 2148 messages amongst the subjects in the non-structured email discussion.
Smith et al (2012) [42]	No data provided regarding use.
Steiner et al (2008) [43]	Secondary analysis of Pierce et al.
Torkamani et al (2014) [48]	No data provided regarding use.
